# Cancer Cell‐Derived Exosomal miR‐500a‐3p Modulates Hepatic Stellate Cell Activation and the Immunosuppressive Microenvironment

**DOI:** 10.1002/advs.202404089

**Published:** 2024-11-22

**Authors:** Yu Zhang, Xin Li, Huiyan Chen, Jiawei Li, Xiaohuan Guo, Yilin Fang, Linjie Chen, Kaiqiang Li, Yi Zhang, Fei Kong, Aodong Chen, Jianxin Lyu, Wei Zhang, Zhen Wang

**Affiliations:** ^1^ Cancer Center Department of Gastroenterology Zhejiang Provincial People's Hospital (Affiliated People's Hospital) Hangzhou Medical College Hangzhou Zhejiang 310014 China; ^2^ School of Ophthalmology and Optometry and Eye Hospital Wenzhou Medical University Wenzhou Zhejiang 325035 China; ^3^ Laboratory Medicine Center Allergy Center Department of Transfusion Medicine Zhejiang Provincial People's Hospital (Affiliated People's Hospital) Hangzhou Medical College Hangzhou 310014 China; ^4^ Department of General Surgery The second affiliated hospital of Zhejiang Chinese Medical University Hangzhou 310015 China

**Keywords:** exosome, hepatic stellate cell, hepatocellular carcinoma, T cell, tumor microenvironment

## Abstract

Hepatocellular carcinoma (HCC) mainly depends on liver fibrosis/cirrhosis, which is regulated by tumor cells and the tumor microenvironment (TME), and is a crucial factor in tumor progression. This study aimed to identify abnormally expressed miR‐500a‐3p in the hepatitis‐cirrhosis‐HCC pathway and explored the roles of miR‐500a‐3p in HCC progression. A clinical cohort of patients with HCC is studied retrospectively. Subsequently, the role of miR‐500a‐3p transported by HCC exosomes in hepatic stellate cell (HSC) activation, hepatoma growth and invasion, and immune cell differentiation is determined by in vitro and in vivo experiments. In clinical tissues, miR‐500a‐3p is significantly enriched in HCC and cirrhosis tissues, and co‐expression of the immune marker CD4 or PD‐L1 significantly correlates with low survival rates in patients. Extracellular miR‐500a‐3p is taken up by HSC and PBMC, which promotes the secretion of the cytokines TGF‐β1 and IL‐10, increases PD‐L1 expression in HSC, and stabilizes PD‐1 expression in PBMC to affect the TME. Moreover, miR‐500a‐3p is associated with CD4^+^ T‐cell exhaustion and Treg differentiation and is significantly associated with increased tumorigenicity in in situ mouse HCC models. Mechanistically, HCC‐derived exosomal miR‐500a‐3p directly influences SOCS2 to regulate the JAK3/STAT5A/STAT5B signaling pathway. MiR‐500a‐3p promotes the growth and migration of HCC through the SOCS2/JAK3/STAT5A/STAT5B axis.

## Graphical abstract

MiR‐500a‐3p, enriched in HCC and cirrhosis tissues, promotes HCC progression by regulating the tumor microenvironment. Exosomal miR‐500a‐3p from HCC cells activates hepatic stellate cells, and enhances cytokine secretion, and Treg differentiation. Mechanistically, miR‐500a‐3p targets SOCS2 to regulate the JAK3/STAT5A/STAT5B pathway, promoting HCC growth and migration.

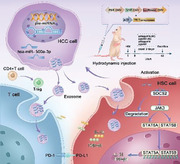

## 1 Introduction

Hepatocellular carcinoma (HCC) accounts for 80% of liver cancers worldwide and is the main type of primary liver cancer.^[^ ^1^ ^]^ Despite current treatment options, including surgical resection, liver transplantation, radiofrequency ablation, and vascular interventional therapy,^[^ ^2^ ^]^ immunotherapy has shown promising prospects in treating advanced HCC.^[^ ^2^, ^3^ ^]^ The development and progression of HCC are complex processes, regulated not only by cancer cells but also by the cellular and non‐cellular components of the tumor microenvironment (TME). Within the TME, the tumor immune microenvironment plays a crucial role in modulating tumor progression, including prevention of tumor immune escape and resistance to drug therapy.^[^ ^3^ ^]^ Recent therapeutic strategies have thus focused on modulating the immune response. Therapeutically blocking T‐cell co‐suppressor molecules, including cytotoxic T‐lymphocyte‐associated protein 4 (CTLA‐4), programmed cell death Receptor 1 (PD‐1), or its ligand (PD‐L1), has shown durable antitumor responses and long‐term remission in various solid tumors.^[^ ^4^ ^]^ Despite promising results of immunotherapies, variable patient responses and the lack of validated predictive biomarkers highlight the urgent need for reliable prognostic tools in HCC treatment.

HCC development is usually triggered by hepatic fibrosis and cirrhosis, both of which are inextricably linked to microenvironmental remodeling and local chronic inflammation.^[^ ^5^ ^]^ Hepatic stellate cells (HSC), specific non‐parenchymal cells located between the hepatic sinusoidal wall and hepatocytes, play a key role in hepatocyte function and liver injury.^[^ ^6^ ^]^ HSCs are normally “quiescent” and undergo significant changes in their biological functions when stimulated by cancer paracrine factors in the TME and phenotypic transformation into highly proliferative tumor‐associated fibroblasts.^[^ ^6^, ^7^ ^]^ Activated HSCs express α‐SMA and release excessive extracellular matrix, including collagens and cellular fibronectin that promote hepatic fibrosis.^[^ ^8^ ^]^ Emerging evidence indicates that HSCs play an important role in cancer initiation, progression, metastasis, and therapy resistance by communicating with cancer cells via exosomes.^[^ ^7^, ^9^ ^]^ Notably, activated HSCs not only serve as central drivers of fibrosis but also contribute to liver immune tolerance by interacting with various immune cells.

Tumor and stromal cells can mutually adapt by secreting soluble factors such as cytokines and chemokines, shaping a microenvironment favorable for tumor growth.^[^ ^10^ ^]^ Exosomes, biologically active lipid‐bilayer nanovesicles serving as novel mediators of intercellular signal communication, play a crucial role in this process.^[^ ^11^ ^]^ Tumor‐derived exosomes can transfer genetic material to other cells, which modulates the phenotype and function of recipient cells, and thus influence tumor progression.^[^ ^12^ ^]^ Among the exosomal cargo, microRNAs (miRNAs) have garnered particular attention due to their stability and functional diversity. Numerous studies have shown that exosomal miRNAs play key roles in tumor‐associated immunosuppression, metabolic reprogramming, and angiogenesis.^[^ ^7^, ^13^ ^]^ For instance, exosomes released by ER‐stressed HCC cells can upregulate PD‐L1 expression in macrophages, subsequently inhibiting T‐cell function via the exosome miR‐23a‐3p‐PTEN‐AKT pathway.^[^ ^13^ ^]^ In addition, Gli1‐induced MIRLET7BHG facilitated HCC by activating HSCs through exosomal SMO to stimulate the hedgehog pathway.^[^ ^7^ ^]^ However, the role of HCC‐derived exosome miRNAs in the immunosuppressive microenvironment, especially its interaction with HSCs and immune cells, should be further explored.

**Scheme 1:**
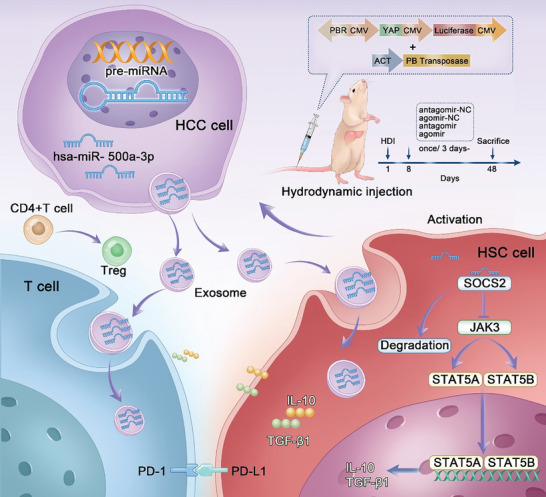
Schematic of the MiR‐500a‐3p‐mediated immunosuppressive effect in HCC autobiography.

This study integrates bioinformatics and experimental approaches to screen abnormally expressed miR‐500a‐3p during hepatitis‐cirrhosis‐HCC transition, focusing on its function and molecular mechanisms in HCC progression and immune microenvironment remodeling. We found that the miR‐500a‐3p/SOCS2/JAK3/STAT5A/STAT5B feedback loop enhances HSC activation and HCC growth and metastasis. Exosomal miR‐500a‐3p released by HCC cells enhanced the immunosuppressive microenvironment by facilitating CD4+ T‐cell exhaustion, regulatory T‐cell differentiation by upregulating PD‐L1 expression in activated HSCs, and PD‐1 expression in PBMCs. Our findings provide novel insights into the mechanisms of miR‐500a‐3p in reshaping the HCC immune microenvironment and provide a theoretical basis for developing miRNA‐targeted immunotherapies (**Scheme** **1**).

## 2 Results

### 2.1 High Levels of miR‐500a‐3p Expression Correlate with Progression and Poor Prognosis for Patients with HCC

**Figure 1:**
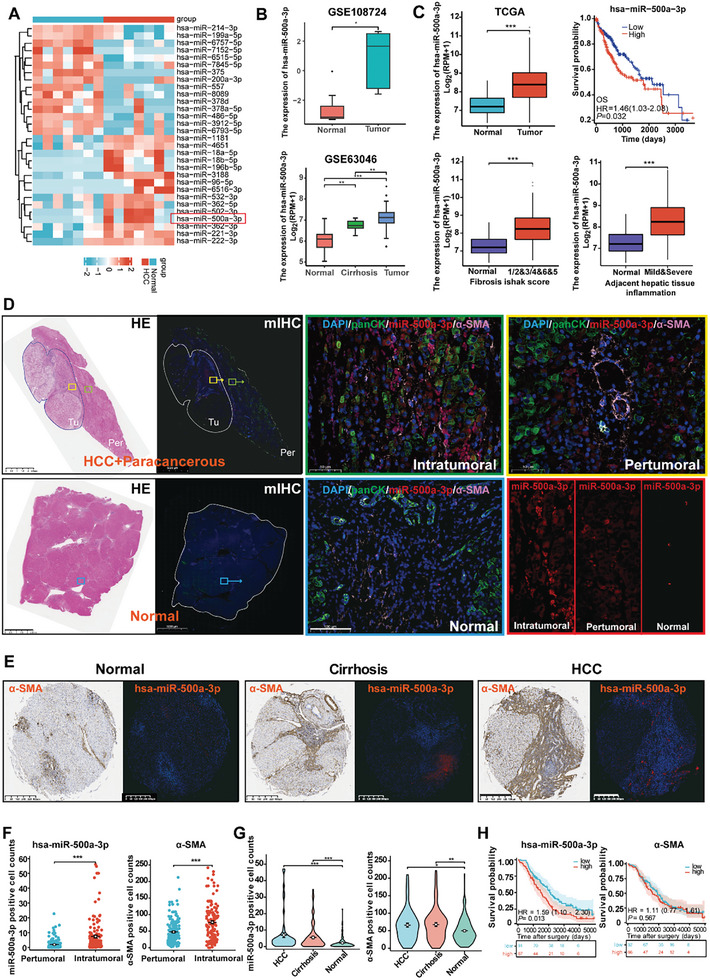
Expression and characterization of miR‐500a‐3p in HCC tissues from public databases and clinical cohorts A,B) Diagram showing the procedure to obtain miR‐500a‐3p from the GEO databases. Heatmaps show the top 30 miRNAs differentially expressed in HCC versus normal tissues in GSE108724 (A). Box diagrams show the miR‐500a‐3p expression level in the tumor, cirrhosis, and normal tissues (B) in the GEO databases (GSE108724 and GSE63046). C) Bioinformatics analysis of miR‐500a‐3p expression using the TCGA database. D) Hematoxylin and eosin (bar value = 2.5 mm) and mIHC (bar value = 5000 and 100 µm) images in the sections show intratumoral (Tu), pertumoral (Per), and normal tissues after co‐expression of α‐SMA (fluorescence pink) and pan‐CK (fluorescence green) with miR‐500a‐3p (fluorescence red). E–G) Expression of miR‐500a‐3p fish probe staining (bar value = 300 µm) and α‐SMA (bar value = 400 µm) in clinical TMA with HCC (n = 158), cirrhosis (n = 69), and normal (n = 43) tissues (bar value = 400 µm). H) Prognostic relationship between miR‐500a‐3p and α‐SMA and clinical patients with HCC (n = 158 intratumoral tissues and n = 112 pertumoral tissues). Data were statistically analyzed using unpaired two‐tailed Student's t‐tests (F), one‐way analysis of variance (G), or Cox regression (H). Data are presented as the mean ± standard deviation, ns > 0.05,^*^ *p* < 0.05, ^**^ *p* < 0.01, ^***^ *p* < 0.001.

We analyzed GSE108724 (HCC vs Normal) and GSE63046 (Tumor vs Cirrhosis vs Normal) datasets obtained from the Gene Expression Omnibus (GEO) database to investigate the potential biomarkers associated with the progression from normal liver tissue through cirrhosis to HCC (**Figure** **1**A,B). The results showed that miR‐500a‐3p was highly expressed in both HCC and cirrhosis tissues compared with normal tissues, and this expression tended to increase with an increase in the degree of tissue heterosexuality (Figure 1B). The Cancer Genome Atlas (TCGA) database indicated high miR‐500a‐3p expression in tumor tissues, which correlated with the patient survival rate (Figure 1C). In addition, miR‐500a‐3p expression was closely related to the liver fibrosis score and hepatic inflammation (Figure 1C), suggesting its role in the hepatitis, cirrhosis, and HCC progression pathway. We conducted immunofluorescence multi‐staining (mIHC) of miR‐500a‐3p, pan‐CK, and α‐SMA (an activated HSC marker) in the tumor, adjacent, and normal tissues from HCC patients. Previous studies have reported that activated HSCs mainly aggregate in regions such as tumor margins, the hepatic sinusoid, tumor stroma, and perivascular areas in HCC tissues.^[^ ^14^, ^15^ ^]^ Consistently, our results demonstrated that miR‐500a‐3p exhibited a significant upregulation in these α‐SMA‐high expressing regions within tumor tissues (Figure S1, Supporting Information). This co‐localization pattern suggests a potential association between miR‐500a‐3p and HSC activation in the HCC microenvironment. Furthermore, we found that miR‐500a‐3p expression showed an increasing trend in normal, para‐cancerous, and tumor tissues (Figures 1D; Figure S2A, Supporting Information), which reinforces its potential role in HCC progression. The clinical relevance of miR‐500a‐3p with activated HSC expression in patients with HCC (Table S1, Supporting Information) was further investigated using Fish and IHC (Figure 1E) to analyze miR‐500a‐3p expression and α‐SMA in a TMA consisting of 158 HCC, 69 cirrhosis, and 43 normal tissues. Notably, miR‐500a‐3p expression levels and α‐SMA were mainly localized in the cytoplasm (Figure 1E) and progressively increased in liver normal, cirrhosis, and cancer tissues (Figure 1F,G). Kaplan–Meier survival analysis showed that miR‐500a‐3p predicted poor prognosis in patients with HCC, whereas α‐SMA showed no difference in predicting prognosis (Figure 1H).

Furthermore, high miR‐500a‐3p expression significantly correlated with α‐SMA in cirrhosis tissues (p = 0.049; Figure S2B, Supporting Information). Multivariate Cox analysis indicated that both miR‐500a‐3p, sex, and tumor size were independent predictors of overall survival (OS) in patients with HCC (Table S2, Supporting Information). Based on multivariate regression analysis, a prognostic nomogram was used to establish the relationship between the variables in the prediction model (Figure S2C, Supporting Information). Overall, these results corroborated the miRNA microarray data, indicating that increased miR‐500a‐3p expression may impact the progression from normal liver to cirrhosis and HCC tissues and correlate with poor prognosis for patients with HCC.

### 2.2 Hsa‐miR‐500a‐3p Facilitated HCC Cell Proliferation, Migration, and Tumor Growth In Vitro and In Vivo

**Figure 2:**
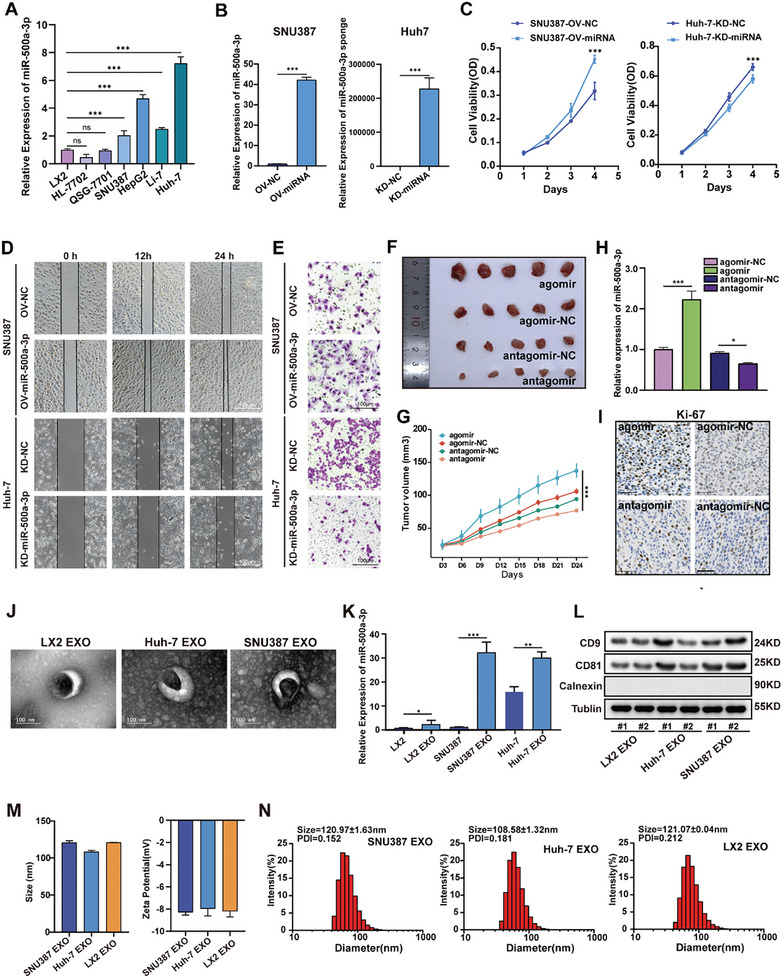
MiR‐500a‐3p facilitated HCC cell proliferation and migration characteristics. A) Determination of miR‐500a‐3p expression in hepatic stellate cells and hepatocellular carcinoma cells using RT‐qPCR (n = 3). B) A lentiviral vector for miR‐500a‐3p‐OV or miR‐500a‐3p‐KD was successfully constructed to transfect SNU387 and Huh‐7, respectively (n = 3). C) The CCK‐8 assay was used to determine the effect of miR‐500a‐3p on the proliferation of SNU387 and Huh‐7 cells (n = 3). D,E) Wound healing assay (D) and transwell assay (E) to detect the effects of miR‐500a‐3p on the migration ability of SNU387 and Huh‐7 cell lines (n = 3). F,G) Representative images (F) and tumor volume of xenograft tumor (G) treated with agomir, agomir‐NC, antagomir‐NC, and antagomir (n = 5 for each group). H) RT‐qPCR was used to determine the miR‐500a‐3p level in each group (n = 3). I) IHC staining determined Ki‐67 positivity in tumors from different groups (bar value = 50 µm). J) Transmission electron microscopy image of HCC‐derived exosomes (n = 3, bar value = 100 nm). K) miR‐500a‐3p expression in hepatic stellate and hepatocellular carcinoma cells and corresponding exosomes (n = 3). L) Exosome markers (CD81, CD9, and Calnexin) in HCC‐derived and HSC‐derived exosomes were detected using Western blot analysis (n = 3). M,N) Nanoparticle tracking analysis of exosomes confirms that more than 95% of the detected particles ranged from 30–150 nm in diameter (n = 3). Data were statistically analyzed using one‐way analysis of variance (A,H,K), unpaired two‐tailed Student's t‐tests (B), or two‐way repeated measures analysis of variance (C, G). Data are presented as the mean ± standard deviation, ns> 0.05,^*^ *p* < 0.05, ^**^ *p* < 0.01, ^***^ *p* < 0.001.

The expression level of miR‐500a‐3p in HCC cell lines (HL‐7702, QSG‐7701, SNU387, HepG2, Li‐7, and Huh‐7) and an HSC line (LX2) was analyzed. As revealed by RT‐qPCR, the miR‐500a‐3p level was higher in HCC cells than in LX2 cells (**Figure** **2**A). Since Huh‐7 cells had the highest miR‐500a‐3p expression and SNU387 cells had a relatively low expression, they were used in subsequent assays; miR‐500a‐3p was overexpressed in SNU387 cells via lentiviral vector transfection and knocked down in Huh7 cells via sh‐miR‐500a‐3p transfection for the subsequent assays (Figure 2B). The CCK8 staining assay revealed significantly increased cell viability with miR‐500a‐3p overexpression in SNU387 cells and reduced viability with miR‐500a‐3p silencing in Huh‐7 cells (Figure 2C). The results of the wound healing assays demonstrated relatively shortened wound healing with miR‐500a‐3p knockdown in Huh‐7 cells and longer wound healing with overexpressed miR‐500a‐3p in SNU387 cells (Figures 2D; Figure S2D, Supporting Information). Furthermore, the Transwell assay demonstrated significantly reduced cell migration in Huh‐7 cells with miR‐500a‐3p depletion and increased cell migration with miR‐500a‐3p overexpression (Figure 2E; Figure S2E, Supporting Information). The results of subcutaneous tumor in nude mice demonstrated that with the injection of antagomir, the tumor growth rate was significantly slower than that of the agomir and control groups (Figure 2F,G), and the miR‐500a‐3p levels were confirmed by RT‐qPCR (Figure 2H). Data from the IHC assay provided similar results, i.e., that miR‐500a‐3p promoted tumor growth, as the positivity of the proliferative marker Ki‐67 was markedly elevated in the agomir group and reduced in the antagomir group (Figure 2I). Based on these data, we concluded that miR‐500a‐3p contributed to cell proliferation and migration in HCC.

### 2.3 HCC‐Derived Exosomes Mediate miR‐500a‐3p Transfer to HSCs

**Figure 3:**
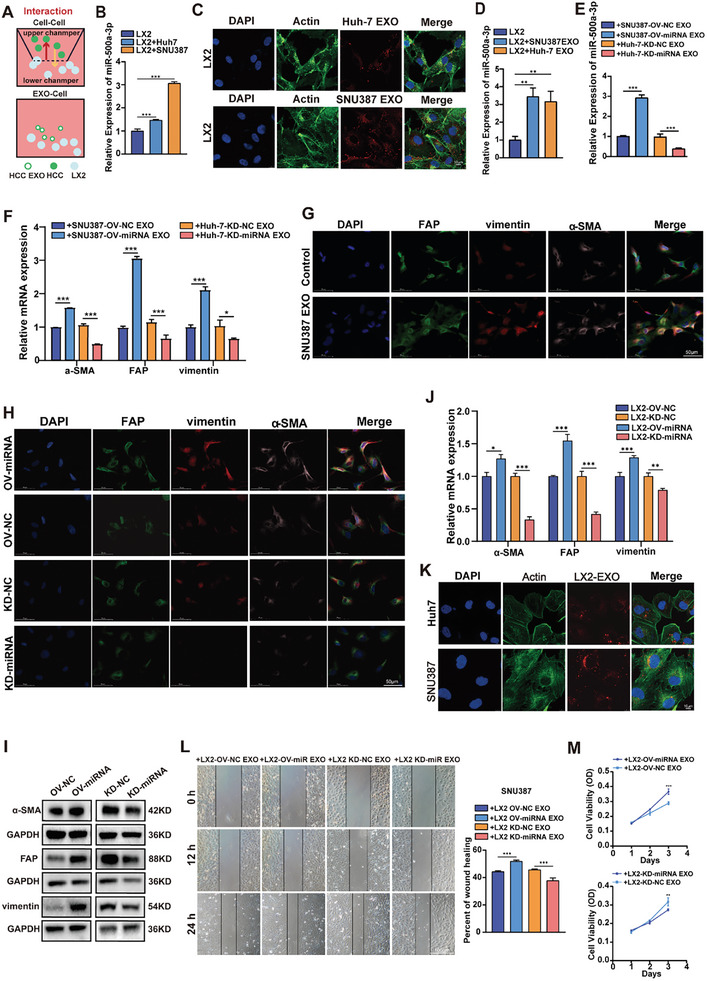
Exosomal miR‐500a‐3p mediates reciprocal activation between HCC cells and HSCs. A) Diagram of LX‐2 cells co‐cultured with HCC cell lines or exosomes. B) Determination of miR‐500a‐3p expression in LX2 and after co‐culture with HCC cells, respectively, using RT‐qPCR (n = 3). C) HCC‐EXO (red) and LX2 cells (green cytoskeleton and blue nuclei) were co‐cultured, and co‐localization was observed using confocal microscopy (bar value = 10 µm). D) Determination of miR‐500a‐3p expression in LX2 cells after co‐culture with HCC exosomes using RT‐qPCR (n = 3). E,F) Effect of HCC exosomes with miR‐500a‐3p‐OV or miR‐500a‐3p‐KD on miR‐500a‐3p expression (E) and hepatic stellate cell activation (F) in LX2 using RT‐qPCR (n = 6). G) Multi‐immunofluorescence staining to determine the effect of SUN387 exosomes on hepatic stellate cell activation (bar value = 50 µm). H–J) Multi‐immunofluorescence staining (H) (bar value = 50 µm), Western blot (I), and RT‐qPCR (J) (n = 3) to detect the effect of miR‐500a‐3p‐OV or miR‐500a‐3p‐KD on LX2 cell activation. K) LX2‐EXO (red) and HCC cells (green cytoskeleton and blue nuclei) were co‐cultured, and co‐localization was observed by confocal microscopy (bar value = 10 µm). L) Wound healing assay to detect the effects of LX2 miR‐500a‐3p‐OV‐ or miR‐500a‐3p‐KD‐derived exosomes on the migration ability of SNU387 cells (n = 3). M) CCK8 assay to detect the effects of LX2 miR‐500a‐3p‐OV‐ or miR‐500a‐3p‐KD‐derived exosomes on the proliferation ability of SNU387 cells (n = 3). Data were statistically analyzed using one‐way analysis of variance or two‐way repeated measures analysis of variance (M). Data are presented as the mean ± standard deviation, ns > 0.05,^*^ *p* < 0.05, ^**^ *p* < 0.01, ^***^ *p* < 0.001.

As exosomes facilitate communication between tumor, mesenchymal, and immune cells, we investigated whether HCC cell‐derived exosomes promote HSC activation. Electron microscopy confirmed that the isolated particles from the cultural supernatants of LX2, Huh‐7, and SNU387 cells were exosomes (Figure 2J). We examined miR‐500a‐3p levels in LX2 cells, HCC cells, and exosomes and found significant upregulation of miR‐500a‐3p in exosomes, indicating their miRNA enrichment capacity (Figure 2K). Furthermore, exosome markers were detected, and Western blotting confirmed high expression of CD81 and CD9, while calnexin was not detected (Figure 2L). Moreover, flow cytometry was used to analyze the size of these exosomes (Figure 2M,N). To determine whether such changes were induced by elevated miR‐500a‐3p in HCC cells, we examined miR‐500a‐3p levels in LX2 cells treated with the indicated HCC cell lines (**Figure** **3**A). Previous studies have reported that HSC could be activated by the cytokine TGF‐β. LX2 cells were co‐cultured with HCC cell lines, or treated with TGF‐β as a positive control. The specific activation marker α‐SMA for activated HSCs was increased under both conditions (Figure S2F, Supporting Information). As expected, miR‐500a‐3p was more upregulated in activated LX2 cells (co‐cultures with HCC lines) than in quiescent LX2 cells (Figure 3B). The influence of HCC exosomes on adjacent HSCs was explored by co‐culturing LX2 cells with the exosomes of SNU387 and Huh7 cells. Dil (red) labeling of HCC exosomes and actin (green) labeling of the cytoskeleton demonstrated that exosomes could be absorbed into LX2 cells (Figure 3C). As expected, miR‐500a‐3p was upregulated in LX2 cells with HCC‐exosomes compared to LX2 cells (Figure 3D). In essence, HCC cells transmit miR‐500a‐3p to LX2 cells via HCC cell‐derived exosomes, which contributes to the complex intercellular communication network within the HCC microenvironment.

### 2.4 MiR‐500a‐3p Mediates Reciprocal Activation between HCC Cells and HSCs via Exosomal Transfer

The study indicated that miR‐500a‐3p expression levels (Figure 3E) and HSC activation markers (α‐SMA, FAP, and vimentin) were gradually boosted in LX2 cells after treatment with HCC exosomes (Figure 3F). Multi‐immunofluorescence staining results demonstrated that LX2 transitioned from a quiescent state to an activated state, with a higher expression of the HSC activation index (vimentin, FAP, and α‐SMA) after co‐culture with SNU387 exosomes (Figures 3G; Figure S1G, Supporting Information). Subsequent assays were performed to evaluate LX2 cell proliferation and migration based on miR‐500a‐3p expression to determine whether such changes were induced by elevated miR‐500a‐3p in HSC cells (Figure S2H, Supporting Information). Transwell assays showed significantly reduced cell migration in LX2 cells with miR‐500a‐3p depletion and increased cell migration with miR‐500a‐3p overexpression (Figures S2I, S1J, Supporting Information). Additionally, the CCK8 assay revealed significantly higher cell viability in LX2 cells with miR‐500a‐3p overexpression and reduced viability with miR‐500a‐3p silencing (Figure S2K, Supporting Information). The mIHC, RT‐qPCR, and Western blot assays showed that miR‐500a‐3p overexpression in LX2 cells significantly increased the expressions of α‐SMA, FAP, and vimentin, while miR‐500a‐3p knockdown decreased the expression of these markers (Figures 3H–J; Figure S1L, Supporting Information). Upon treatment with HCC cell‐derived exosomes, activated LX2 cells may exhibit certain characteristics that indirectly promote tumor progression when compared with quiescent LX2 cells. We further investigated whether activated HSCs could influence HCC progression. Immunofluorescence staining results demonstrated that LX2 exosomes can be phagocytosed into HCC cells (Figure 3K). Subsequent wound healing assays indicated that HSC‐derived exosomes with high miR‐500a‐3p levels significantly enhanced HCC cell migration compared to exosomes with low miR‐500a‐3p levels (Figure 3L). The CCK8 assay indicated that LX2‐derived exosomes with miR‐500a‐3p‐OV markedly increased the proliferation of HCC cells, while HSC‐derived exosomes with miR‐500a‐3p‐KD inhibited cell growth compared to the NC groups (Figure 3M). Our findings reveal a bidirectional exosomal miR‐500a‐3p‐mediated crosstalk between HCC cells and HSCs, which promotes HSC activation and enhances HCC progression.

### 2.5 MiR‐500a‐3p Activates HSCs by Targeting SOCS2 and Modulating the JAK3/STAT5A/STAT5B Axis

**Figure 4:**
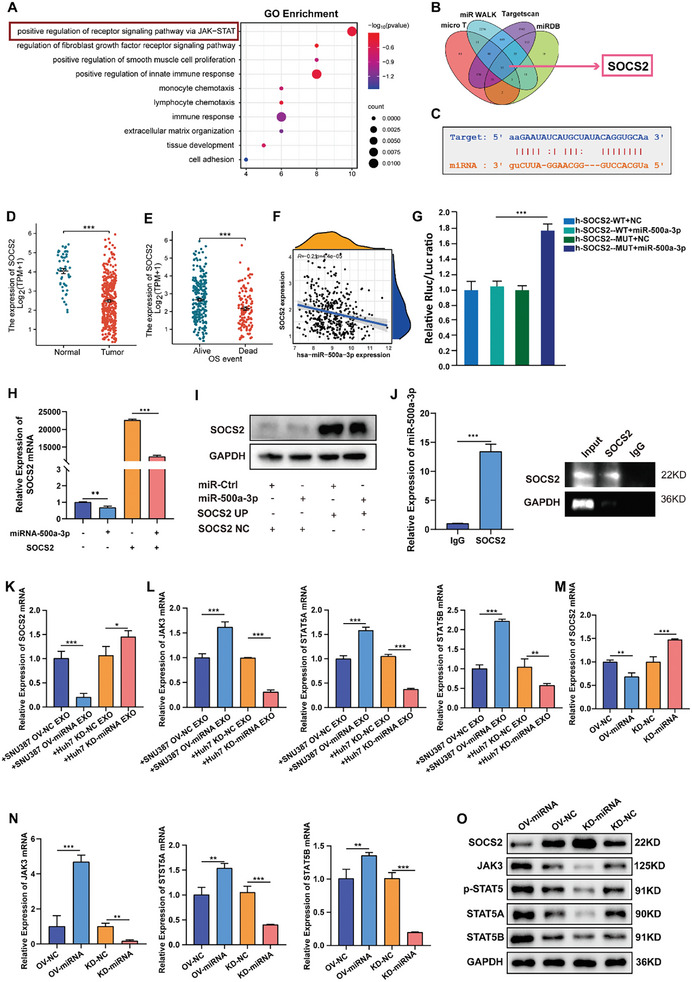
MiR‐500a‐3p activates HSCs via the SOCS2/JAK3/STAT5A/STAT5B axis. A) RNA‐seq analysis was performed on the three groups of LX2 miR‐500a‐3p‐OV and the NC group, and GO analysis was performed on the differential genes obtained (n = 3). B) Target mRNA with potential binding sites for miR‐500a‐3p as predicted by micro T, miRwalk, Targetscan, and miRDB. C) Putative binding sites of miR‐500a‐3p in SOCS2. D) SOCS2 expression in tumor and normal tissues in the TCGA LIHC cohort. E) Relationship between SOCS2 and overall survival of patients with HCC in the TCGA cohort. F) The scatter diagram indicated that miR‐500a‐3p expression positively correlated with SOCS2 in the TCGA LIHC cohort. G) Luciferase activity of the SOCS2 dual‐luciferase reporter vector (WT or MUT) in HEK293T cells co‐transfected with miR‐500a‐3p (n = 3). H,I) RT‐qPCR and Western blot analyses of the relative levels of SOCS2 expression in LX2 cells after transfection with miR‐500a‐3p mimics (n = 3). J) After a RIP assay had been performed with a SOCS2 plasmid, RT–qPCR indicated significant miR‐500a‐3p enrichment compared to negative controls (n = 3). K,L) Relative levels of SOCS2/JAK3/STAT5A/STAT5B mRNA in LX2 cells after co‐culture with Huh‐7 miR‐500a‐3p‐KD or SNU387 miR‐500a‐3p‐OV‐derived exosomes (n = 6). M–O) RT‐qPCR (M,N) and Western blot (O) analyses of the relative levels of SOCS2/JAK3/STAT5A/STAT5B expression in LX2 cells after transfection with miR‐500a‐3p‐KD or miR‐500a‐3p‐OV (n = 6). Data were statistically analyzed using unpaired two‐tailed Student's t‐tests (J) or one‐way analysis of variance. Data are presented as the mean ± standard deviation, ns > 0.05,^*^ *p* < 0.05, ^**^ *p* < 0.01, ^***^ *p* < 0.001.

We explored the upstream mechanism of miR‐500a‐3p in activated HSCs. First, we performed RNA‐seq analysis on LX2 cells with overexpressed mir‐500a‐3p and mir‐500a‐3p‐NC to determine the function and pathway of mir‐500a‐3p in HSC activation. We found that miR‐500a‐3p plays a significant role in the JAK‐STAT signaling pathway, the fibroblast growth factor receptor signaling pathway, and the immune response in the GO function of LX2 cells (**Figure** **4**A). We then searched for the mRNAs targeting miR‐500a‐3p in four databases: microT, miR Walk, Targetscan, and miRDB. Through the Venn diagram intersection, we found that 32 mRNAs were predicted to bind to miR‐500a‐3p in the four databases (Figure 4B). High‐stringency screening of the four databases indicated that PPP2R5E, CAMK4, SOCS2, SOCS4, SOCS5, and SOCS6 target miR‐500a‐3p. Members of the SOCS family are cytokine‐induced negative regulators of cytokine receptor signaling by regulating the activation of the JAK/STAT pathway. Combined with the previous results of RNA‐Seq GO analysis of LX2 cells, we focused on key genes within the JAK‐STAT signaling pathway. Moreover, RT‐qPCR analysis indicated that miR‐500a‐3p overexpression negatively regulated SOCS2 expression in LX2 cells. However, the mRNA expression levels for PPP2R5E, CAMK4, SOCS4, SOCS5, and SOCS6 did not significantly correlate with miR‐500a‐3p levels (Figure S3A, Supporting Information). The potential binding sites between miR‐500a‐3p and SOCS2 were obtained from the Targetscan database (Figure 4C). Furthermore, SOCS2 expression was significantly higher in normal tissues than in tumor tissues (Figure 4D) and negatively correlated with the T Stage in patients with HCC (Figure S3B, Supporting Information). Moreover, Kaplan–Meier analysis revealed that SOCS2 could predict OS (Figure 4E) and disease‐free survival (Figure S3C, Supporting Information) for patients with HCC in the investigated TCGA databases. There was a significant negative correlation between SOCS2 and miR‐500a‐3p expressions in HCC tissues (Figure 4F). The dual‐luciferase reporter assay also demonstrated that a miR‐500a‐3p mimic significantly reduced the luciferase activity of the SOCS2 3′‐UTR WT reporter but not that of the mutant SOCS2 3′‐UTR reporter (Figure 4G). In LX2 cells transfected with miR‐500a‐3p‐OV, miR‐500a‐3p overexpression significantly decreased SOCS2 levels, and re‐introduction of SOCS2 abolished the miR‐500a‐3p‐induced decrease in SOCS2 (Figure 4H,I). The RIP assay revealed significant enrichment of miR‐500a‐3p in the anti‐SOCS2 group compared to the anti‐IgG group (Figure 4J). This suggests that miR‐500a‐3p acts as an endogenous sponge directly targeting SOCS2. Furthermore, we elevated miR‐500a‐3p expression in HCC cells and found that upregulating miR‐500a‐3p significantly reduced SOCS2 expression in exosomes‐activated LX2 cells (Figure 4K). Next, we added HCC cell‐derived exosomes to LX2 cells and examined the JAK/STAT signaling pathway. We found that the JAK3, STAT5A, and STAT5B levels were markedly increased in LX2 cells when activated by HCC cell‐derived exosomes (Figure 4L). To determine whether the JAK3/STAT5A/STAT5B pathway was regulated by miRNA‐500a‐3p in activated HSCs, we analyzed the expression of SOCS2, JAK3, STAT5A, and STAT5B using RT‐qPCR and Western blot after overexpressing or reducing miRNA‐500a‐3p levels. The results showed that miR‐500a‐3p overexpression in LX2 cells reduced the level of the negative regulatory factor SOCS2 (Figure 4M) but increased JAK3 expression and its down‐stream proteins STAT5A and STAT5B compared with the control group, along with the phosphorylation level of STAT5 (Figure 4N,O). Furthermore, miRNA‐500a‐3p suppression promoted JAK3, STAT5A, and STAT5B expression and activated STAT5 phosphorylation. These results confirmed that HCC exosome‐derived miRNA‐500a‐3p regulates the activation of HSCs through the SOCS2/JAK3/STAT5A/STAT5B pathway.

### 2.6 Exosomal miR‐500a‐3p Promotes Immunosuppression in HCC by Regulating the PD‐L1/PD‐1 Axis and Treg Expansion

**Figure 5:**
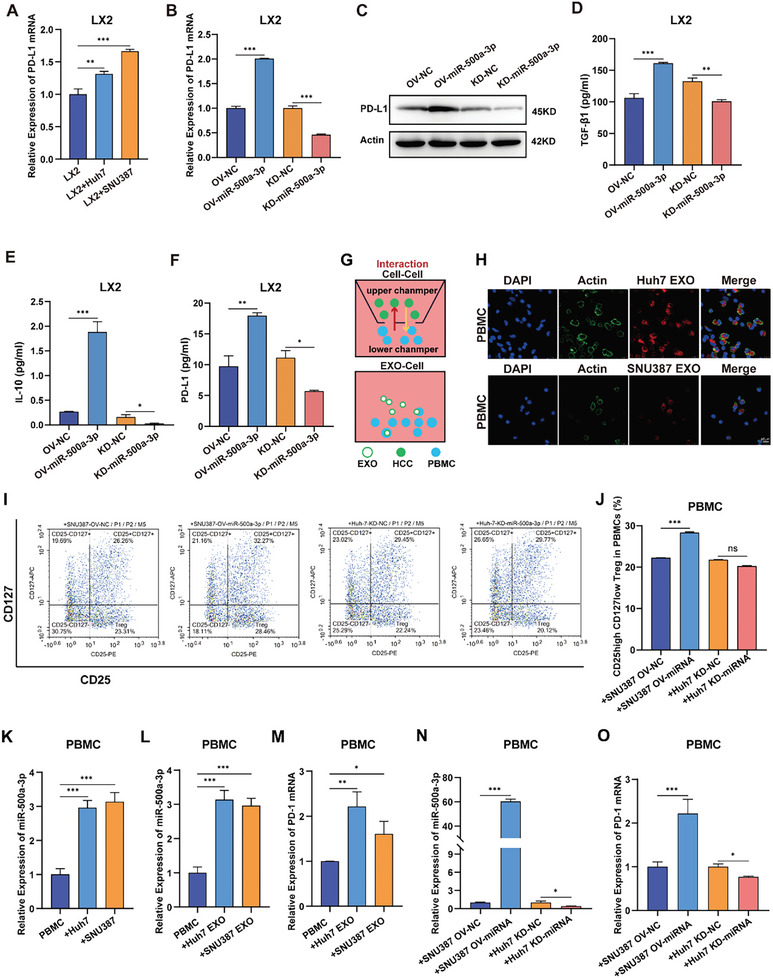
MiR‐500a‐3p regulates PD‐L1 expression in HSCs and PD‐1 expression in PBMCs. A) RT‐qPCR to determine PD‐L1 expression in LX2 cells or after co‐culture with HCC cells (n = 3). B,C) RT‐qPCR (B) and Western blot (C) analyses of the relative levels of PD‐L1 expression in LX2 cells after transfection with miR‐500a‐3p‐KD or miR‐500a‐3p‐OV (n = 3). D–F) The content of TGF‐β1, IL‐10, and PD‐L1 in the supernatant of hepatic stellate cells transfected with the miR‐500a‐3p‐KD or miR‐500a‐3p‐OV was detected by ELISA (n = 3). G) Diagram of PBMC cells cocultured with HCC cell lines or exosomes. H) HCC‐EXO (red) and PBMC (green cytoskeleton and blue nuclei) were co‐cultured, and co‐localization was observed by confocal microscopy (bar value = 10 µm). I,J) Flow cytometry was used to detect the proportion of CD127^low^CD25^high^ T cells in PBMC after co‐culturing with HCC cells with miR‐500a‐3p‐KD or miR‐500a‐3p‐OV (n = 3). K) RT‐qPCR to determine the expression of miR‐500a‐3p in PBMC or after co‐culturing with HCC cells. L,M) RT‐qPCR to determine the expression of miR‐500a‐3p (L) and PD‐1 (M) in PBMC or after co‐culturing with HCC‐derived exosomes (n = 3). N,O) RT‐qPCR to determine the expression of miR‐500a‐3p (N) and PD‐1 (O) in PBMC after co‐culturing with HCC cells with miR‐500a‐3p‐KD or miR‐500a‐3p‐OV (n = 3). Data were statistically analyzed using a one‐way analysis of variance. Data are presented as the mean ± standard deviation, ns >0.05, ^*^ *p* < 0.05, ^**^ *p* < 0.01, ^***^ *p* < 0.001.

Previous research has underscored the TME as a crucial space for interactions between tumor and immune cells. Zhao et al. highlighted the integral role of HSCs in promoting HCC progression, partly through their immune regulatory activities.^[^ ^14^ ^]^ STAT5 expression was significantly and positively correlated with T‐cell enrichment, cytolytic activity, and PD‐L1 expression in HCC.^[^ ^16^ ^]^ In addition, we found a close relationship between miR‐500a‐3p and PD‐L1 expression in cirrhosis tissues (Figure S3D, Supporting Information), suggesting that miR‐500a‐3p may play a role in the immune TME of HCC. Next, we analyzed the correlation between STAT5A, STAT5B, and PD‐L1 expression in the TCGA database; we found that both STAT5A and STAT5B were positively correlated with PD‐L1 in HCC tissue (Figure S3E, Supporting Information). Moreover, PD‐L1 expression significantly increased in LX2 cells co‐cultured with HCC CMs (**Figure** **5**A). To determine whether such changes in PD‐L1 expression were induced by elevated miR‐500a‐3p in LX2 cells, we assessed PD‐L1 levels in LX2 cells with miR‐500a‐3p overexpression or knockdown. RT‐qPCR and Western blot results showed increased PD‐L1 expression in LX2 cells with miR‐500a‐3p overexpression and decreased expression in cells with miR‐500a‐3p knockdown (Figure 5B,C). Furthermore, we analyzed cytokines secreted by HSC cells, and ELISA showed increased secretion of the immunosuppressive cell stimulators TGF‐β1, IL‐10, and PD‐L1 in the miR‐500a‐3p overexpression group and decreased secretion in the knockdown group (Figure 5D–F), suggesting that miR‐500a‐3p may mediate the formation of HSC‐mediated immunosuppressive cytokines. We further investigated the effect of exosomal miR‐500a‐3p on T cells. Exosomes isolated from HCC cell supernatant were incubated with PBMC (Figure 5G). For exosome tracking, miR‐500a‐3p‐derived exosomes labeled with the red fluorescent dye Dil were efficiently absorbed by PBMCs (Figure 5H). Co‐culturing of miR‐500a‐3p‐overexpressing HCC cells increased the proportion of CD127^low^CD25^high^ T cells (Figure 5I,J). Moreover, we detected the expression of common markers associated with Treg exhaustion on the cell surface (for example, PD‐1) using RT‐qPCR. The results showed significant increases in miR‐500a‐3p and PD‐1 levels in PBMC after co‐culture with HCC exosomes (Figure 5K–M). PD‐1 mRNA levels significantly increased in miR‐500a‐3p overexpression groups and decreased in miR‐500a‐3p knockdown groups (Figure 5N,O). These results suggest that miR‐500a‐3p in HCC exosomes promotes the secretion of immunosuppressive factors and the expression of immune checkpoint PD‐1/PD‐L1 in the HCC TME.

### 2.7 In Vivo Validation of the role of miR‐500a‐3p in HCC Progression and Immune Regulation

**Figure 6:**
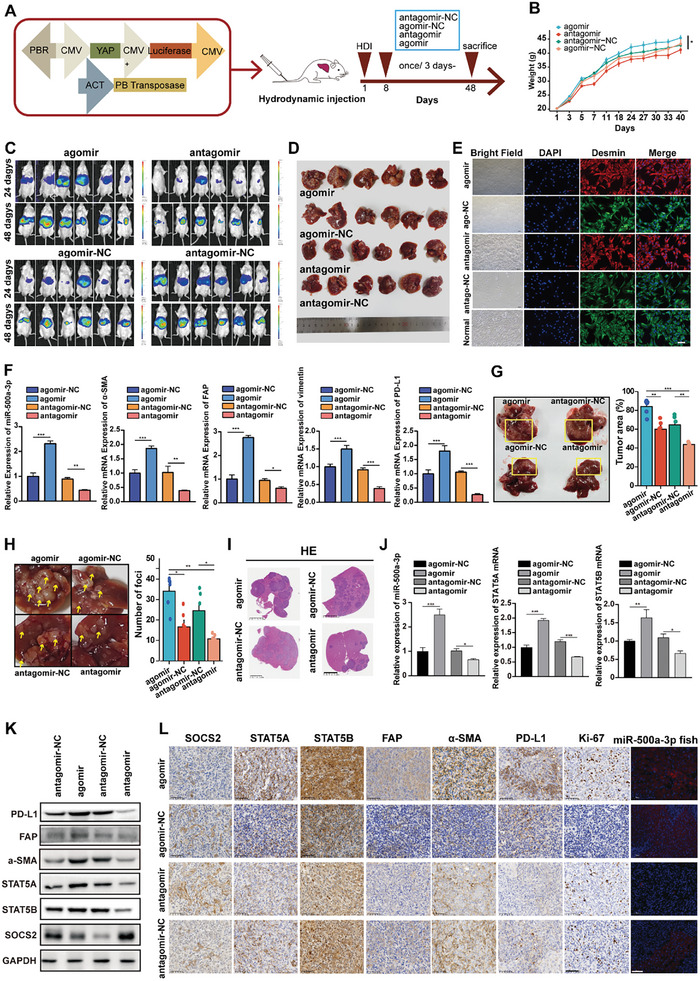
MiR‐500a‐3p promotes HCC growth and HSC activation via the SOCS2/STAT5/PD‐L1 axis in mouse models. A) Diagram of the procedure to establish PB‐YAP‐HDI orthotopic HCC models and the agomir miR‐500a‐3p, agomir‐NC, antagomir‐NC, and antagomir miR‐500a‐3p treatments. B) Growth curves for mouse weight were measured twice a week after every injection of PB‐YAP in ICR mice. C) IVIS imaging to detect the growth of in situ liver tumors at 24 and 48 days in the four groups (n = 6 for each group). D) Representative images of orthotopic liver tumor treated with agomir, agomir‐NC, antagomir‐NC, and antagomir (n = 6 for each group). E) The morphology of primary HSCs extracted from ICR mice was visualized by white light and immunofluorescence microscopy (bar value = 100 µm). F) RT‐qPCR to determine the levels of miR‐500a‐3p, HSC activation indicators (α‐SMA, FAP, and vimentin), and PD‐L1 in the four groups (n = 3). G–I) Tumor area (G), tumor foci (H), and HE staining images (bar value = 2.5 mm) (I) in the four groups. The data for the tumor area are shared with Figure 6D. J) RT‐qPCR to determine the levels of miR‐500a‐3p and STAT5A/STAT5B expression in the four groups (n = 3). K) Western blotting to determine the levels of SOCS2/STAT5A/STAT5B expression, HSC activation indicators (α‐SMA and FAP), and PD‐L1 in the four groups (n = 3). L) Representative images of IHC staining of mouse tumors revealed the effects of miR‐500a‐3p from HSCs on the SOCS2/ STAT5A/STAT5B axis, the activation of α‐SMA and FAP, the proliferation index Ki‐67, and PD‐L1 markers (bar value = 50 µm). Data were statistically analyzed using one‐way analysis of variance or two‐way repeated measures analysis of variance (B). Data are presented as the mean ± standard deviation, ^*^ *p* < 0.05, ^**^ *p* < 0.01, ^***^ *p* < 0.001.

Having established the influence of miR‐500a‐3p on PD‐L1 expression in cell lines, we confirmed this regulatory effect in an in situ HCC ICR mouse model. After inducing an in situ HCC tumor with the PB‐YAP plasmid in ICR mice, we sought to validate the effects of miR‐500a‐3p agomir or antagomir on tumor growth in vivo. In line with the finding that miR‐500a‐3p is a vertebrate‐specific miRNA, a miR‐500a‐3p seed‐binding site in the SOCS2 3′UTR was conserved across vertebrates, indicating its functional significance (Figures S4A,B, Supporting Information). According to IVIS and FISH probe staining results, both agomir and antagomir were enriched in in situ HCC (Figures S4C,D, Supporting Information). ICR mice in the miR‐500a‐3p agomir, antagomir, and their negative control groups were injected via the tail vein every three days to grow for 48 days (**Figure** **6**A). The data showed that the mice in the mir‐500a‐3p overexpression group had higher weights than those in the mir‐500a‐3p knockdown groups (Figure 6B). Next, we extracted primary HSCs from mouse liver tissues. First, white light microscopy and fluorescence microscopy were used to determine the morphology of primary HSCs (Figure 6E). RT‐qPCR (Figure 6F) and multi‐immunofluorescence staining (Figure S5A, Supporting Information) were used to verify the expression of HSC activation indexes in each group. The in situ HCC results showed that in the agomir group, HSCs were more significantly activated than in the control group, and the expression of PD‐L1 was increased. However, in the antagomir group, HSCs activation was reversed, and PD‐L1 expression decreased (Figure 6F; Figure S5A, Supporting Information). The role of miR‐500a‐3p in promoting the progression of HCC was further confirmed in the mouse model; the tumor area (Figure 6G) and number of tumor foci (Figure 6H,I) in the agomir group were significantly higher than those in the antagomir and the control groups. RT‐qPCR and Western blotting showed that mir‐500a‐3p and STAT5A/B mRNA expression was significantly higher in the agomir group and partially downregulated in the antagomir group compared to the control group (Figure 6J,K). IHC analysis of the in situ HCC tissues demonstrated significant enhancement in SOCS2, STAT5A, STAT5B, α‐SMA, FAP, Ki‐67, and PD‐L1 in specimens with higher miR‐500a‐3p expression and suppression in specimens with lower miR‐500a‐3p expression, while the IHC expression of SOCS2 showed an opposite trend (Figure 6L; Figure S5B, Supporting Information). Huh‐7 cells were used to construct a xenograft tumor model of HCC to verify the role of miR‐500a‐3p in HCC (Figures S4D, S5C, Supporting Information). The results for miR‐500a‐3p in subcutaneous tumors in nude mice were consistent with those in situ HCC models. These findings demonstrate a robust positive correlation between miR‐500a‐3p and the SOCS2/STAT5A/STAT5B/PD‐L1 signaling axis in vivo.

### 2.8 MiR‐500a‐3p Influenced CD4+ T‐Cell Exhaustion and Treg Differentiation in HCC

**Figure 7:**
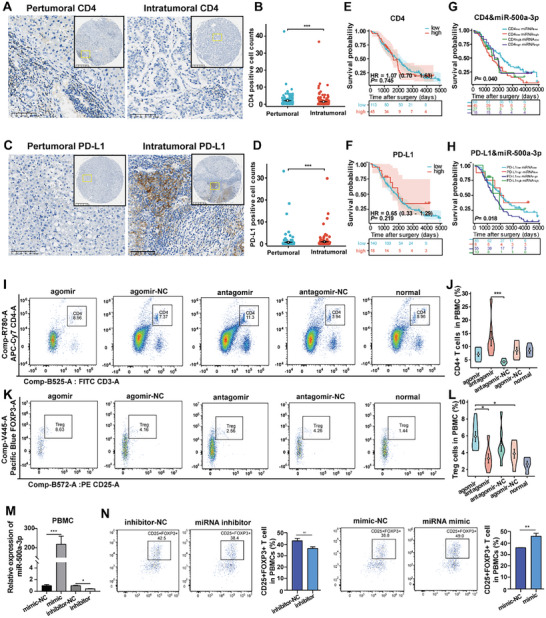
MiR‐500a‐3p influenced CD4+ T‐cell exhaustion, Treg differentiation, and HCC Prognosis. A–D) IHC staining and the scatter diagram of the expression level in intratumoral and peritumoral CD4 (A,B) and PD‐L1 (C,D) in patients with HCC (n = 158 intratumoral tissues and n = 112 peritumoral tissues; bar value = 100 and 400 µm). E,F) Kaplan–Meier survival curves showing the OS for intratumoral CD4 (E) and PD‐L1 (F) expression based on a tissue microarray including 158 patients with HCC. G,H) Kaplan–Meier survival curves showing the OS for intratumoral CD4 (G) and PD‐L1 (H) co‐expression with miR‐500a‐3p based on a tissue microarray including 158 patients with HCC. I–L) Flow cytometry was used to detect the proportion of CD4+ T cells (I,J) and regulatory T cells (K,L) in the agomir, agomir‐NC, antagomir, antagomir‐NC, and normal groups in in situ HCC ICR mouse models (n = 6 for each group). M) RT‐qPCR to determine the expression of miR‐500a‐3p in PBMC or after transfection with miR‐500a‐3p mimic or inhibitor (n = 3). N) Flow cytometry was used to detect the proportion of CD25+FOXP3+ T cells in human PBMC after transfection with miR‐500a‐3p mimic, inhibitor, and control (n = 3). Data were statistically analyzed using unpaired two‐tailed Student's t‐tests (B,D,N), Cox regression (E–H), or one‐way analysis of variance (J,L,M). Data are presented as the mean ± standard deviation, ^*^ *p* < 0.05, ^***^ *p* < 0.001.

IHC was used to analyze the expression of CD4 and PD‐L1 in the clinical HCC TMA and the impact on HCC prognosis. The results showed that CD4 was highly expressed in paracancerous tissue (**Figure** **7**A,B), while PD‐L1 was highly expressed in tumor tissue (Figure 7C,D). Kaplan–Meier survival analysis indicated no difference between CD4 and PD‐L1 in predicting HCC prognosis (Figure 7E,F). However, upon stratifying CD4 and PD‐L1 expression with miR‐500a‐3p, we found a significant correlation with HCC prognosis (Figure 7G,H). Furthermore, we analyzed the effect of miR‐500a‐3p on the activity and function of CD4 + T cells and Treg in ICR HCC mice. Blood samples from ICR mice treated with miR‐500a‐3p agomir and antagomir were analyzed using flow cytometry. The results indicated that miR‐500a‐3p antagomir significantly enhanced the activity of CD4^+^ T cells, whereas miR‐500a‐3p agomir decreased their activity (Figure 7I,J). Moreover, the proportion of Treg in the miR‐500a‐3p agomir group was significantly higher than in the miR‐500a‐3p antagomir group (Figure 7K,L

), and the secretion of cytokine TGF‐β1 in agomir group was significantly higher than that in antagomir group (Figure S4E, Supporting Information). To confirm whether miR‐500a‐3p participates in T cell differentiation, we transfected miR‐500a‐3p mimic into human PBMCs (Figure 7M) and detected the proportion of CD25+FOXP3+ T cells by flow cytometry. Compared with the control group, the proportion of Treg in the over‐expressed miR‐500a‐3p group significantly increased (Figure 7N). These results indicate that miR‐500a‐3p contributes to the formation of an immunosuppressive microenvironment in HCC by promoting CD4+ T‐cell exhaustion and Treg differentiation.

## 3 Discussion

HCC development is regulated by both tumor cell factors and the surrounding microenvironment. The tumor immune microenvironment, comprising various cell types, forms an immunosuppressive milieu through intercellular communication, thus participating in tumor genesis, development, and metastasis.^[^ ^17^ ^]^ Emerging evidence indicates that activated HSCs in the HCC tumor milieu may accelerate hepatocyte neoplastic transformation. Stationary HSCs mainly maintain liver homeostasis and prevent HCC development, while activated myofibroblast HSCs (myHSC) promote tumor cell proliferation and malignant transformation.^[^ ^6^ ^]^ The balance between different HSC subtypes, such as cytokine‐ and growth factor‐expressing HSCs (cyHSCs) and myHSCs, may be critical in determining the pro‐ or anti‐tumorigenic effects of the HCC microenvironment.^[^ ^6^ ^]^ Our study revealed a significant upregulation of miR‐500a‐3p expression in HCC tissues compared to normal liver tissues, with predominant overexpression in α‐SMA+ myofibroblast regions. This aligns with the results of previous studies that demonstrated that activated HSCs are mainly distributed in areas critical for HCC progression, such as tumor margins and stroma.^[^ ^14^, ^15^ ^]^ These findings suggest that miR‐500a‐3p may exert its pro‐tumorigenic effects by modulating the activation and function of HSCs, particularly of the myHSC subpopulation, in the HCC microenvironment.

To further investigate the role of miR‐500a‐3p in HCC, we used RNA sequencing to profile miRNA expression in HCC, liver cirrhosis, and normal tissues from the GEO database and identified miR‐500a‐3p as a significantly upregulated miRNA in each group. Several studies have highlighted the pro‐tumor effect of miR‐500a, showcasing its elevated expression in human cancers (such as chronic lymphocytic leukemia) and non‐neoplastic diseases (such as endometriosis). Jiang et al. found that miR‐500a‐3p promoted the characteristics and tumorigenicity of cancer stem cells in HCC through the STAT3 signaling pathway.^[^ ^18^ ^]^ Consistent with these findings, we observed miR‐500a‐3p upregulation in HCC tissues and cells and that miR‐500a‐3p overexpression was associated with poorer OS and disease‐free survival in patients with HCC. Our functional experiments confirmed that miR‐500a‐3p directly promotes the activation of HSCs and enhances their pro‐tumorigenic properties, providing a mechanistic link between miR‐500a‐3p expression and HSC activation. These findings reveal a novel pathway through which miR‐500a‐3p may orchestrate the complex interplay within the HCC microenvironment.

Intercellular communication within the TME is important in tumor onset, development, and distant metastasis.^[^ ^19^ ^]^ As newly discovered intercellular communication tools in recent years, exosomes play an important role in tumor progression.^[^ ^20^, ^21^ ^]^ Tumor cells can transfer genetic material to surrounding tumor cells by secreting exosomes, altering their biological functions, and promoting tumor growth and metastasis.^[^ ^22^ ^]^ Studies have demonstrated the diverse effects of exosomes on stromal cells. For instance, bladder cancer was found to induce fibroblasts to differentiate into tumor‐associated fibroblasts through exosomes, resulting in elevated IL‐6 secretion.^[^ ^23^ ^]^ Similarly, a recent study found that gastric cancer transmits miR‐27a to fibroblasts through exosomes to transform them into tumor‐associated fibroblasts, thus promoting gastric cancer growth and metastasis.^[^ ^24^ ^]^ In the context of HCC, our study revealed that exogenous miR‐500a‐3p and HCC‐derived exosomal miR‐500a‐3p promoted HSC proliferation, migration, and activation. Furthermore, endogenous miR‐500a‐3p enhanced HCC proliferation and invasion by activating HSCs. Hence, miR‐500a‐3p acts as a signaling molecule bridging HCC‐to‐HSC cell communication. In addition, these results suggest the functional properties of miR‐500a‐3p and the triggering of hepatocarcinogenesis during HCC development. MiR‐500a‐3p was a critical signal transducer in the TME cellular network when preferentially exposed to cancer cells via the transport of HCC‐derived exosomes.

The crosstalk between mRNAs can be mediated by miRNAs, and our study identified miR‐500a‐3p as a key regulator of the *SOCS2* gene. Through bioinformatics and experimental validation, we demonstrated that miR‐500a‐3p directly binds to the highly conserved 3′UTR of SOCS2, suggesting its functional significance. Our findings reveal that miR‐500a‐3p influences HCC progression by competitively binding SOCS2, a process largely dependent on HCC‐derived exosome‐mediated miRNA transport. Remarkably, even low concentrations of exosomes from miR‐500a‐3p‐overexpressing groups effectively transferred miRNA to HSCs, significantly altering downstream target gene expression and highlighting the pivotal role of exosomal‐mediated miRNA in shaping the TME. SOCS2 protein, as a major negative regulator, plays an important role in the carcinogenesis of various tumors by regulating cytokine signaling through the JAK/STAT axis.^[^ ^25^ ^]^ In HCC, downregulation of SOCS2 is associated with tumor progression and poor prognosis.^[^ ^25^ ^]^ Our RNA‐seq analysis confirmed that miR‐500a‐3p overexpression in HSCs activated the JAK/STAT pathway, while further mRNA and protein analysis indicated that SOCS2 downregulation led to significantly enhanced activity of JAK3, STAT5A, STAT5B, and p‐STAT5. This was accompanied by upregulation of HSC activation‐related proteins and increased levels of pro‐inflammatory and pro‐tumoral mediators. Given the importance of the JAK3/STAT5A/STAT5B pathway in inflammatory responses and the role of the peritumoral inflammatory environment in promoting intrahepatic metastasis, we hypothesize that this signaling pathway may promote intrahepatic metastasis by converting pro‐inflammatory responses into tumor progression. This provides a new theoretical basis for HCC pathogenesis and development, offering new ideas and strategies for tumor immune intervention.

In the TME, exosomes serve as crucial mediators of the communication between tumor and immune cells, often contributing to the formation of an immunosuppressive microenvironment. Studies have shown that both immune and tumor cell‐derived exosomes can modulate HCC progression and immune escape.^[^ ^26^, ^27^ ^]^ Specifically, tumor‐derived exosomes can suppress T‐lymphocyte functions, affecting both CD8+ and CD4+ T cells, and disrupting the Th17/Treg balance.^[^ ^26^ ^]^ In the present study, we demonstrated that HCC‐derived exosomal miR‐500a‐3p could be absorbed by PBMCs, leading to the upregulation of PD‐1 expression and an increase in the proportion of Tregs, which contributed to an immunosuppressive TME. Tregs, characterized by high CD25 and Foxp3 expression, mediate immune tolerance through cell contact mechanisms or secretion of inhibitory cytokines such as IL‐10 and TGF‐β.^[^ ^28^ ^]^ Similarly, nasopharyngeal carcinoma‐derived exosomes have been shown to promote the conversion of CD4+CD25‐ T cells into CD4+CD25+ T cells, accompanied by increased secretion of IL‐10 and TGF‐β, which mediates tumor immune escape.^[^ ^29^ ^]^ Consistent with these findings, our ELISA results revealed that the uptake of HCC‐derived exosomes by HSCs significantly increased the secretion of IL‐10 and TGF‐β, which may further promote T‐cell differentiation in the TME. The importance of miR‐500a‐3p in modulating the immune microenvironment was further supported by our in vivo experiments, in which miR‐500a‐3p overexpression in the in situ mouse liver cancer model led to a significant increase in the proportion of tumor‐infiltrating T cells.

In addition to its effects on PBMCs, we observed that HCC‐derived exosomal miR‐500a‐3p could upregulate the expression of PD‐L1 in activated HSC cells and PD‐1 in PBMCs. Notably, our analysis of clinical tissues revealed that combined miR‐500a‐3p and PD‐L1 expression could serve as a potential prognostic biomarker for HCC patients. The PD‐1/PD‐L1 signaling pathway is a key mechanism of tumor immune evasion, in which PD‐L1 binding to PD‐1 leads to inhibition of T‐cell proliferation and cytokine secretion, as well as induction of T‐cell apoptosis and exhaustion.^[^ ^30^ ^]^ This finding further underscores the immunosuppressive role of miR‐500a‐3p in the HCC microenvironment. Our study reveals the pivotal role of HCC‐derived exosomal miR‐500a‐3p in HCC progression and immunosuppressive TME formation. MiR‐500a‐3p exerts pro‐tumorigenic effects through multiple mechanisms, highlighting the complex interactions between tumor, stromal, and immune cells in the HCC microenvironment. However, our study has some limitations that warrant further investigation. Future research should aim to elucidate the specific mechanisms underlying the effects of HCC‐derived exosomal miR‐500a‐3p on T‐cell function and differentiation, as well as explore the feasibility and efficacy of targeting miR‐500a‐3p in combination with current immunotherapeutic strategies.

## 4 Conclusion

This study highlights the important role of HCC‐derived exosomal miR‐500a‐3p in intercellular communication, which may be responsible for HSC activation and tumor aggressiveness via the downstream SOCS2/JAK3/STAT5A/STAT5B regulatory loop. We demonstrated that miR‐500a‐3p in HCC‐derived exosomes could induce the transformation of HSCs into tumor myoblasts, release immunosuppressive cytokines, and stimulate PD‐L1 expression, thereby exerting immunosuppressive functions and ultimately promote HCC growth and metastasis. Our findings demonstrate that HSCs are key modulators of an immunosuppressed HCC microenvironment, and this modulation is associated with decreased CD4^+^ T‐cell infiltration and upregulation of Treg expression. Notably, miR‐500a‐3p combined with PD‐L1 predicted HCC outcomes, hinting at their potential as prognostic indicators for HCC and immunotherapy efficacy.

## 5 Experimental Section

### Ethics Approval and Consent to Participate

This study was conducted in accordance with the Declaration of Helsinki and approved by the Institutional Review Board of Zhejiang Provincial People's Hospital [ZJPPHEC 2023I (076)]. The blood samples of healthy individuals were collected at Zhejiang Provincial People's Hospital; all participants provided written informed consent to participate. Pathological paraffin tissues and clinical features of patients with HCC were studied retrospectively. The requirement for informed consent in this respect was waived owing to the retrospective nature of the study and the anonymous handling of patient data. All animal experiments were performed according to the protocols approved by the Laboratory Animal Management and Ethics Committee of Zhejiang Provincial People's Hospital (IACUC‐A20230508007).

### Patients and Specimens

Specimens were collected from 158 patients who were pathologically confirmed to have HCC after R0 curative hepatectomy from January 2007 to December 2018. None of the patients had received prior anticancer therapy or had other malignancies. Clinical information (including sex, age, tumor size, pathological stage, lymph node status, vascular invasion, cancer thrombosis, nerve invasion, and underlying cirrhotic disease) was extracted from the hospital case system and conducted subsequent assessments up to June 2022. The clinicopathological characteristics of patients are summarized in Table S1 (Supporting Information).

### Hematoxylin and Eosin (H&E) and IHC Staining

The tissue samples were cut into conventional 5 µm sections and subsequently subjected to H&E staining for further analysis. IHC was performed on tissue microarrays (TMAs) according to the manufacturer's instructions (Invitrogen, Carlsbad, CA, USA). Briefly, in accordance with previous protocols, dewaxed and rehydrated tissue sections were pretreated with an EDTA antigen repair solution (Beijing, Origene, China) at pH 8 and heated for 15 min. Sections were cooled, decolorized in phosphate buffer, and incubated for 20 min at 18—25 °C.^[^ ^31^ ^]^ Negative and positive controls were included for each marker in each experiment. Immunostaining was performed on TAMs using anti‐α‐SMA (HA600032; Huabio), anti‐CD4 (ab183685; Abcam), and anti‐PD‐L1 (GT228007; Genetech) antibodies. Tumors from mouse flanks were fixed in formalin for at least 24 h and embedded in paraffin. Subsequently, immunostaining and H&E staining were performed on mouse tissues using anti‐α‐SMA, anti‐SOCS2 (ab109245; Abcam), anti‐JAK3 (ab45141; Abcam), anti‐STAT5A (ab32043; Abcam), anti‐STAT5B (ab178941; Abcam), anti‐STAT5 (phospho Y694; ab32364; Abcam), and anti‐PD‐L1 (GT228007; Genetech) antibodies.

### Multiplex‐Immunostaining Assays

The cell samples were fixed with paraformaldehyde for 10 min and permeated with 0.3% triton X‐100 membrane‐breaking solution for 20 min. The tissue was uniformly covered with 3% BSA in the chemical ring for more than 30 min. Repeated rounds of labeling were performed as follows: primary antibody incubation, secondary antibody incubation, and fluorescent dye reaction solution reaction. The sections of primary antibody vimentin (dilution ratio 1:800), Fap (dilution ratio 1:100), and α‐SMA (1:2000) were placed flat in a wet box at 4 °C overnight, and HRP hypersensitive goat and rabbit universal secondary antibody (HKI0029; Haoke) were incubated in the following sequence, respectively. Flare570 red (HKI0015; Haoke), Flare520 green (HKI0014; Haoke), and Flare690 powder (HKI0017; Haoke) were used to label the above 3 indexes fluorescently, and finally 4′,6‐diamidino‐2‐phenylindole (DAPI) was used to re‐stain the nucleus (HKI0005; Haoke). Immunofluorescence was visualized by a microscope slide scanner (Pannoramic SCAN; 3DHISTECH Ltd. Hungary).

### Survival and Correlation Analysis

In the clinical cohort of our hospital, the R software (version 4.2.1) was used to analyze the correlation between miR‐500a‐3p expression and clinicopathological variables using the “stats” package. The “survival” package was used to test the proportional risk hypothesis and fit survival regression, and the results were visualized using the “survminer” and the “ggplot2” packages. The correlation between miR‐500a‐3p and α‐SMA, CD4, and PD‐L1 expression was analyzed using the Spearman method, and the results were visualized using the “ggplot2” package.

### Cell Lines and Treatment

### Cell Lines and Treatment—Exosome Isolation

Exosomes were isolated using the high‐speed centrifugation method. Briefly, cells were cultured in an exosome‐free serum, and the cell supernatant was collected after 72 h. The supernatant was centrifuged at 300 × *g* for 10 min and then at 2000 × *g* and 4 °C for 10 min to remove cell debris and extract the supernatant; finally, the supernatant was centrifuged at 10000 × *g* for 30 min to remove large vesicles. Then, ultracentrifugation was performed at 110000 × *g* for 70 min; the supernatant was discarded, and the exosome‐containing pellet was retained. Exosome precipitates were then resuspended in 20 mL of phosphate‐buffered saline (PBS) and filtered using a 0.22 µm filter before recollection via ultracentrifugation at 110000 × *g* for 70 min.

### Cell Lines and Treatment—Exosome Identification

The isolated exosomes were diluted with 1× PBS, and their particle size was measured using a nanoparticle tracking analyzer (NTA) using ZetaView PMX 110 (Particle Metrix, Meerbusch, Germany) and the corresponding ZetaView 8.04.02 software. The exosomes were used for RNA/protein extraction or cell treatment. Exosomes were quantified via the BCA method and then stored at −80 °C or directly used in subsequent experiments.

### Cell Lines and Treatment—Exosome Fluorescent Labeling

The purified exosomes were labeled using the Dil red fluorescent labeling kit (Sigma) following the manufacturer's protocol. Exosomes (100 µL) were resuspended in 0.5 mL of buffer C and mixed with 5 µL of Dil dye solution. After incubation for 5 min, 2 mL of exosome‐free serum was added to seal off the Dil dye solution for 2 min. The 100 µL exosome‐Dil mixture was then transferred to a confocal chamber and incubated for 2–4 h under dark conditions; 100 µL of 1× Hoechst was then added to stain for 10 min, followed by two washes with PBS. Finally, the uptake of exosomes by HCC cells was observed under a microscope.

### Transmission Electron Microscopy

The exosome was dropped on a copper mesh and negatively stained with 2% phosphotungstic acid for 2 min. If the adsorption was more obvious in the copper mesh, pure water was added to the surface, quickly absorbed, and cleaned several times. After drying, the images were observed and photographed using a transmission electron microscope (FEI Tecnai G2 Spirit, Thermo Scientific, USA).

### RNA Fluorescence In Situ Hybridization (FISH) and Co‐Expression Analysis

A Cy3‐labeled specific probe for miR‐500a‐3p was designed and synthesized by GenePharma; the signals were detected using a FISH Kit (GenePharma, China) according to the manufacturer's instructions. The TMAs prepared from formalin‐fixed tissues of HCC patients were digested with proteinase K at 37 °C for 20 min. After prehybridization, slides were hybridized with the miR‐500a‐3p FISH probe CAGAA+TCCT+TGCCCAGGTGCA+T at 37 °C overnight. The hybridization solution was absorbed and discarded. Each slice was washed with a preheated 100 µL post‐hybridization water solution for 15 min and washed with PBS once. Nuclei were counterstained with 4,6‐diamidino‐2‐phenylindole (DAPI; GenePharma, China). Confocal images were captured using the Zeiss AIM software and a Zeiss LSM 700 confocal microscope system (Carl Zeiss Jena, Oberkochen, Germany). The in situ hybridization reaction for the miRNAs was performed, followed by IHC detection of Pan‐Cytokeratin (pan‐CK; ER1914‐74; Huabio) and α‐SMA in the same section. Pan‐CK was used to identify epithelial cells, and DAPI was used to stain the nucleus. The section was then analyzed using the Nuance computer system, which separates each colorimetric base signal as a different fluorescent color and then mixes them to determine if there is co‐expression.

### Cell Cultures and Cell Transfection

HCC cell lines (SNU387 and Huh7) and HSCs (LX2) were obtained from Procell Life Technology Co., LTD (Wuhai, China) and authenticated using Short Tandem Repeat DNA profiling. Huh7 and LX2 lines were cultured in DMEM (Thermo Fisher Scientific, Waltham, MA, USA), while SNU387 cells were cultured in RPMI 1640 (HyClone, Logan, UT, USA), all supplemented with 1% penicillin/streptomycin (Invitrogen) and 10% FBS (Thermo Fisher Scientific), at 37 °C under 5% CO_2_.

### Cell Cultures and Cell Transfection—Plasmid Construction, Lentivirus Construction, Inhibitor, and Mimic Experiments

*SOCS2* cDNA was cloned into the pcDNA3.1(+) vector and verified through sequencing. MiR‐500a‐3p mimic inhibitors were purchased from RiboBio (Guangzhou, China). HBLV‐ZsGreen‐PURO negative control, HBLV‐HSA‐Mir‐500A‐3P‐Zsgreen‐Puro, and HBLV‐hsa‐miR‐500a‐3p sponge‐ZsGreen‐PURO viruses were purchased from Hanbio (Shanghai, China). The sequences were as follows (sense strand): miR‐500a‐3p mimics, 5′‐AUGCACCUGGGCAAGGAUUCUG‐3′; NCs, 5′‐UUUGUACUACACAAAAGUACUG‐3′; miR‐500a‐3p inhibitors, 5′‐CAGAAUCCUUGCCCAGGUGCAU‐3′; and NC inhibitors: 5′‐CAGUACUUUUGUGUAGUACAAA‐3′. Cells were transfected using Lipofectamine 3000 reagent (Invitrogen, Carlsbad, CA, USA) according to the manufacturer's instructions.

### Western Blot Analysis

Cells were collected, and the proteins were extracted from the cells, as described earlier. The enhanced BCA protein assay kit (Beyotime, China) was used to quantify the protein concentration. An equal amount of protein was separated via sodium dodecyl sulfate‐polyacrylamide gel electrophoresis (SDS‐PAGE) and then transferred onto a polyvinylidene fluoride (PVDF) membrane (Millipore, Bedford, MA, USA). The membrane was then incubated overnight at 4 °C with corresponding antibodies. Subsequently, a developer was prepared at a ratio of 1:1. The membrane was soaked in the developer for 1 min in the absence of light and then exposed for imaging in a multicolor fluorescence chemiluminescence instrument.

### RNA Extraction and qRT‐PCR

Total RNA was extracted from cultured cells and exosomes using MolPureTRIeasyPlus total RNA kits (Shanghai Yisen, China). The mRNA expression was detected using the PrimeScript RT Reagent Kit and SYBR Premix Ex Taq (Takara Bio, Inc., Kyoto), with GAPDH serving as an internal control. The bulge‐loopTM miRNA qRT‐PCR primer set (one RT primer and a pair of qPCR primers per set) specific to miR‐500a‐3p was designed by RiboBio (Guangzhou, China). The qPCR primers are listed in Table S3 (Supporting Information). Additionally, an all‐in‐one microRNA qRT‐PCR assay kit (RiboBio, Guangzhou, China) was used to detect miRNA expression, with U6 serving as the control. Each experiment was performed in triplicate according to the manufacturer's protocol. Final data were analyzed using the 2^−ΔΔCT^ method.

### RNA Sequencing (RNA‐seq) Analysis

RNA was isolated and purified from the total samples using TRIzol (Invitrogen, CA, USA) according to the manufacturer's protocol. The quantity and purity of the total RNA were assessed using the NanoDrop ND‐1000 spectrophotometer (NanoDrop, Wilmington, DE, USA). The integrity of RNA was confirmed using a Bioanalyzer 2100 (Agilent, CA, USA) device and agarose electrophoresis.

The captured mRNA was fragmented under high temperatures using the NEBNext Magnesium RNA Fragmentation Module (item number E6150S, USA). Subsequently, cDNA was synthesized from the segmented RNA using Invitrogen SuperScript II Reverse Transcriptase (No. 1,896,649, CA, USA) to form a library with a fragment size of 300 ± 50 bp. Finally, the Illumina Novaseq 6000 system (LC‐Biotechnologies CO., Ltd. Hangzhou, China) was used to perform double‐end sequencing in the PE150 mode, following standard procedures.

### Dual‐Luciferase Reporter Assay

Cells were co‐transfected with pmiR‐RB‐Report plasmids containing the wild‐type or mutant *SOCS2* 3′‐UTR and miRNA mimics using Lipofectamine 3000 (Invitrogen, Carlsbad, CA, USA). After 48 h, luciferase activity was determined using a Dual‐Luciferase Reporter Assay Kit (Promega, Madison, WI, USA) according to the manufacturer's instructions. Mutant luciferase reporter genes were generated via PCR‐based site‐specific mutagenesis, and a Firefly luciferase signal was used for normalization.

### RNA‐Binding Protein Immunoprecipitation and qRT‐PCR (RIP)

An RNA Immunoprecipitation Kit (GEENSEED, Guangdong, China, No. P0101) was used for RIP. In total, 1 × 10^7^ LX2/miR‐500a‐3p cells (per immunoprecipitation) were collected, according to the detailed specifications of the RIP Kit.

### Cell Viability Assay

For the CCK8 assay, 100 µL of CCK‐8 working solution (with a ratio of 1:9 CCK‐8 reagent to medium) was added to a 96‐well plate. The cells were then incubated at 37 °C for 1 h, and the absorbance at 450 nm was detected using an enzyme marker. The absorbance was measured at the same time points after 24, 48, and 72 h, and statistical analysis was performed to calculate cell viability.

### Cell Migration and Cell Scratch Assays

For migration assays, after infiltrating the Transwell for 24–48 h, cells were fixed and stained with 0.1% crystal violet. For scratch assays, cells were incised, incubated in 500 µL of serum‐free medium for 24–48 h, and observed under a microscope. The cells on the lower surface were photographed and counted using the ImageJ software.

### Hydrodynamic Injection

Male ICR mice aged 4 weeks, purchased from Shanghai SLAC Laboratory Animal Company, were injected with a mixture containing 50 µg of total transposon plasmids and 10 µg of transposase‐expressing plasmids diluted in sterile Ringer's solution at a volume equal to 10% of their body weights.^[^ ^32^ ^]^ The mixture was injected through the tail vein within 5—7 s.^[^ ^32^ ^]^

### Drug Administration

According to the experimental design, mice were randomly divided into the following groups: miR‐500a‐3p OV (agomir 5 µg g^−1^; n = 6), miR‐500a‐3p OV‐NC (agomir‐NC 5 µg g^−1^ n = 6), miR‐500a‐3p KD (antagomir 10 µg g^−1^; n = 6), and miR‐500a‐3p KD‐NC (antagomir‐NC 10 µg g^−1^; n = 6). Treatment for mice in the miR‐500a‐3p antagomir, agomir, or their respective scrambled sequence control (GenePharma, Shanghai, China) groups commenced 7 days after establishing the YAP‐induced mouse model. The agomir and antagomir solutions were diluted with PBS and injected via the tail vein into the mice at a dose of 5 and 10 µg g^−1^ twice a week for 40 days, respectively. The negative control consisted of a scrambled sequence of antagomir or agomir. After the tumors were visible, the tumor growth rate was monitored by measuring tumor diameters and using the IVIS‐100 system (Caliper Life Sciences, Boston, MA, USA) every month with a Vernier caliper. The tumor growth curve was recorded accordingly. After the mice were euthanized, the liver tissues were dissected with six different sections with 50 microns in depth. Tumor areas were measured using the ImageJ software. The samples were prepared for H&E staining and the number of foci was counted under a light microscope.

### Xenograft Experiments

A subcutaneous transplantation tumor model of the huh7 cell line was established. Male nude mice aged 4 weeks were divided into four groups with five mice per group. Tumor formation was between 100 mm^3^ 7 days after the injection of 10 million cells into the left armpit (plus matrix glue). Antagomir, antagomir NC, agomir, and agomir‐NC (3OD/per mouse, once every 2 days, for 14 days) were injected at multiple points around the tumor. The volume of subcutaneous tumors and the weight of mice were observed once every 3 days. Twenty‐four days after injection with huh7 cells, the mice were euthanized, and their tumors were biopsied.

### Primary Hepatic Stellate Cell Isolation

After anesthesia, 1×PBS (pH7.4) containing 50 UI ml^−1^ heparin sodium without Ca^2+^ and Mg^2+^ was preheated at 37 °C for liver perfusion. Then, 0.05% collagenic enzyme was used to alternately perfuse the liver from the hepatic portal vein and inferior vena cava. The mouse liver and the liver capsule were removed to obtain the whole liver cell suspension. Centrifugation was performed at 400 rpm for 10 min, and the precipitated cells were turned into hepatenchymal cells and then centrifuged again. The supernatant was discarded, and the precipitated cells were re‐suspended with 4 mL of 1×PBS. Moreover, 3 mL of 50% Percoll, 4 mL of 25% Percoll, and 2 mL of cell suspension were added and centrifuged at 900 g for 20 min. After centrifugation, the middle cell layer was carefully absorbed and transferred to another centrifuge tube for re‐suspension and centrifugation. The cells were transferred into a T‐25 culture bottle and cultured in a 37 °C incubator for 60 min. The unattached cells were transferred into another culture bottle for culture. The liver stellate cells were cultured in this bottle. Primary HSCs were cultured in a medium (Hunan Fenghui Biotechnology Co., Ltd, Cat NO. sc2023072501).

### PBMC Isolation

Human PBMCs were isolated and purified from healthy donor peripheral blood using the Easy‐Sep Direct Human PBMC Isolation Kit (STEMCELL, Cat. No.19654). Mouse PBMCs were isolated using a mouse PBMC isolation solution kit (Solarbio, Cat. No. P6340). For PBMC activation and proliferation, human PBMCs were seeded into 24‐well plates, and 5 µg mL^−1^ CD3 (BD Biosciences, San Jose, CA, United States of America) and 4000 U mL^−1^ recombinant human IL‐2 were added and incubated for 48 h. The exosomes or miRNA mimics or inhibitors were transfected into preactivated PBMCs using a human T‐cell transfection kit (Lonza).

### Enzyme‐Linked Immunosorbent Assay

The concentrations of IL‐10, TNF‐β1, and PD‐L1 produced by HSCs were measured using IL‐10, TNF‐β1, and PD‐L1 ELISA kits (Liankebio, China; Cat numbers EK110/2‐96, EK981‐96, and EK1261HS‐96) following the manufacturer's guidelines.

### Flow Cytometric Analysis

For flow cytometric analysis, 100 µL of Stain Buffer (BSA; No. 554656) was used to prepare a single‐cell suspension (10^6^ cells), added LIVE/DEAD Fixable Viability Stain 510 (BD, Cat. No. 564406), incubated them at 25 °C without light for 15 min, and then added 1 µg of Fc Block reagent for sealing. Subsequently, cell surface fluorescent antibodies (such as CD45, CD4, and CD25) were added and stained in a dark box for 15 min. Next, the membranes were broken and fixed using the Transcription Factor Buffer Set (BD, Cat. No. 562574) from the membrane breaking kit and incubated at 4 °C for 45 min in the dark. Then, the endonuclear index FOXP3 staining antibody was added, gently mixed and incubated at 4 °C for 45 min in a dark environment. Finally, the cells were resuspended in 400 µL of stain buffer. The data were analyzed using flow cytometry.

### Fluorescence‐Activated Cell Sorting Antibodies

The antibodies for FACS included PerCP Rat Anti‐Mouse CD45 (30‐F11; BD, Cat. No. 557235), APC‐Cy7 Rat Anti‐Mouse CD4 (GK1.5; BD, Cat. No. 552051), PE Rat Anti‐Mouse CD25 (3C7; BD, Cat. No. 553075), FITC Hamster Anti‐Mouse CD3e (145‐2C11; BD, Cat. No. 553061), FOXP3 Monoclonal (FJK‐16s; Thermo, Cat. No. 404‐5773‐82), and Purified Rat Anti‐Mouse CD16/CD32 (Mouse BD Fc Block; BD, Cat. No. 553141) antibodies.

### Statistical Analysis

Continuous variables were presented as the mean ± standard deviation. Student's *t*‐test was used to compare the groups. One‐way analysis of variance was used to compare multiple groups. Categorical data were analyzed using the chi‐squared and Fisher's exact probability tests. The “ggplot2” package of the R software (version 4.2.1) was used for differential analysis, while the “survival” package was used for Cox regression analysis. Nomogram‐related models were constructed and visualized using the “rms” package. All mathematical analyses of the data were performed using GraphPad Prism 8.0, and *p* < 0.05 was considered statistically significant.

### Inclusion and Diversity Statement

The authors support inclusive, diverse, and equitable conduct of research.

## Conflict of Interest

The authors declare no conflict of interest.

## Author Contributions

Y.Z., X.L., H.C., and J.L. contributed equally to this work. Y.Z. and X.L. performed conceptualization. Y.Z., J.L., H.Y.C. performed methodology. K.L. used software. Y.Z., F.K., J.L. performed validation. H.C., A.C. J.L. did formal analysis. Z.W., Y.Z., W.Z. did funding acquisition. J.L. performed investigation. Y.Z., L.C., X.L. did resources. H.C., X.G. did data curation. Y.Z., X.L., Y.F., J.L., H.C. wrote the original draft. J.L., W.Z., Z.W. wrote the review and did editing. Y.Z., H.C., X.L., F.K. performed visualization. J.L., W.Z., Z.W. did supervision. J.L., W.Z., Z.W performed project administration.

## Supporting information



Supporting Information

## Data Availability

The data that support the findings of this study are available from the corresponding author upon reasonable request.
