# Supplementary material for: Cancer Cell‐Derived Exosomal miR‐500a‐3p Modulates Hepatic Stellate Cell Activation and the Immunosuppressive Microenvironment
Source: Adv Sci (Weinh). 2024 Nov 22;12(2):2404089. doi: 10.1002/advs.202404089 (PMC11727405; doi:10.1002/advs.202404089)

Cancer cell-derived exosomal miR-500a-3p modulates hepatic stellate cell activation and the immunosuppressive microenvironment

*Yu Zhang^1#^, Xin Li^3#,^ Huiyan Chen^2#^, Jiawei Li^3#,^ Xiaohuan Guo^2^, Yilin Fang^2^, Linjie Chen^2^, Kaiqiang Li^2^, Yi Zhang^2^, Fei Kong^2^, Aodong Chen^3^, Jianxin Lyu^2*^, Wei Zhang^4*^, Zhen Wang^2*^*

1 Cancer Center, Department of Gastroenterology, Zhejiang Provincial People's Hospital (Affiliated People's Hospital, Hangzhou Medical College), Hangzhou, Zhejiang, China 310014.

2 Laboratory Medicine Center, Allergy Center, Department of Transfusion Medicine, Zhejiang Provincial People's Hospital (Affiliated People's Hospital), Hangzhou Medical College, Hangzhou, 310014, China

3 School of Ophthalmology and Optometry and Eye Hospital, Wenzhou Medical University, Wenzhou, Zhejiang, China

4 Department of General Surgery, The second affiliated hospital of Zhejiang Chinese Medical University, Hangzhou 310015, China

#These authors contributed equally

*Corresponding authors:

E-mail:; wangzhen@hmc.edu.cn; zhangweils1968@163.com; ljx@hmc.edu.cn

**
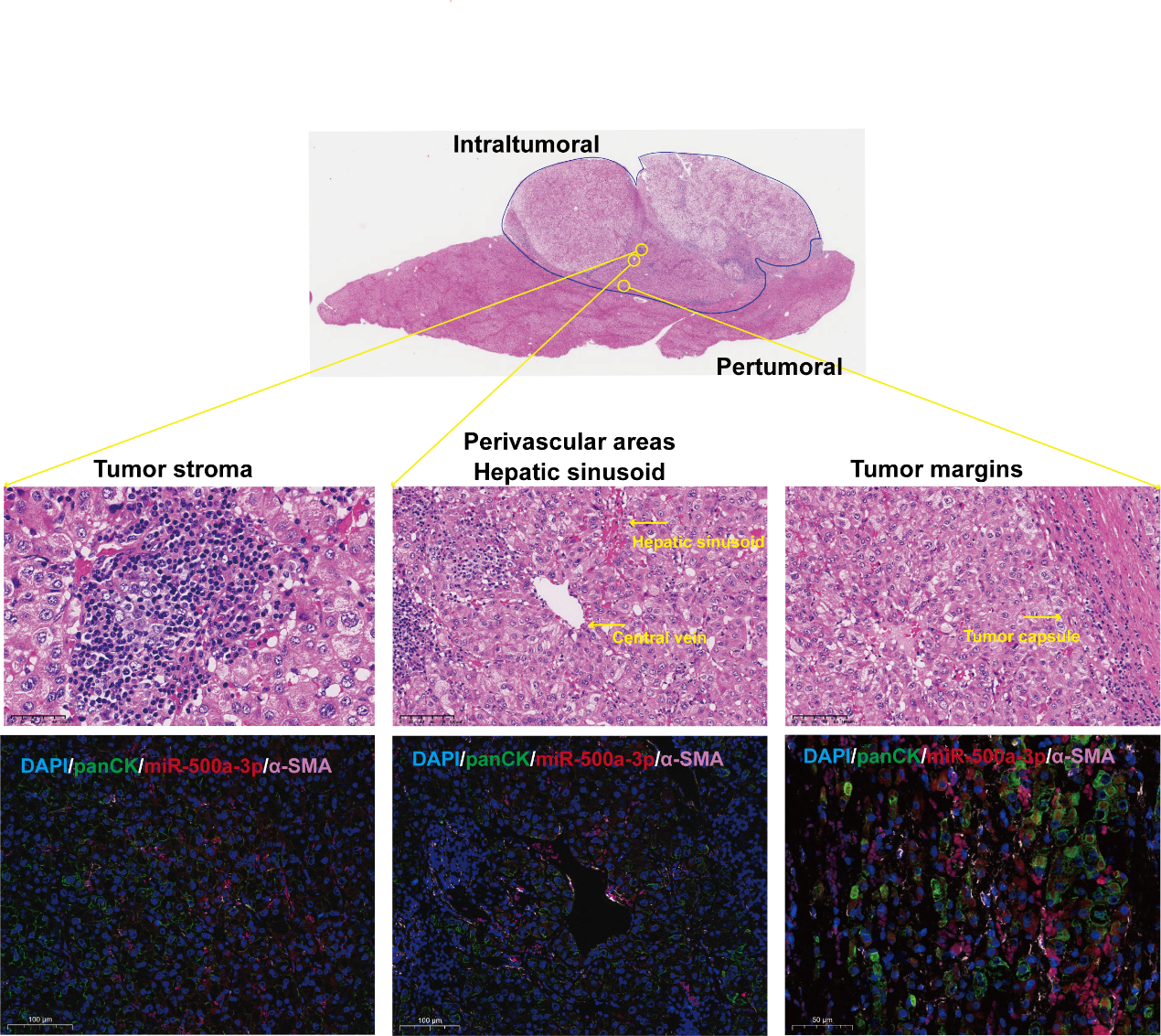
Figure S1.** Co-expression patterns of miR-500a-3p, pan-CK, and α-SMA in different regions of HCC tissues. Representative images of immunofluorescence multi-staining show the co-localization of DAPI (nuclei), pan-CK (epithelial cells), miR-500a-3p, and α-SMA (activated hepatic stellate cells) in the tumor stroma, perivascular areas, hepatic sinusoid, and tumor margins within the intratumoral regions of HCC tissues (bar value = 100 μm).


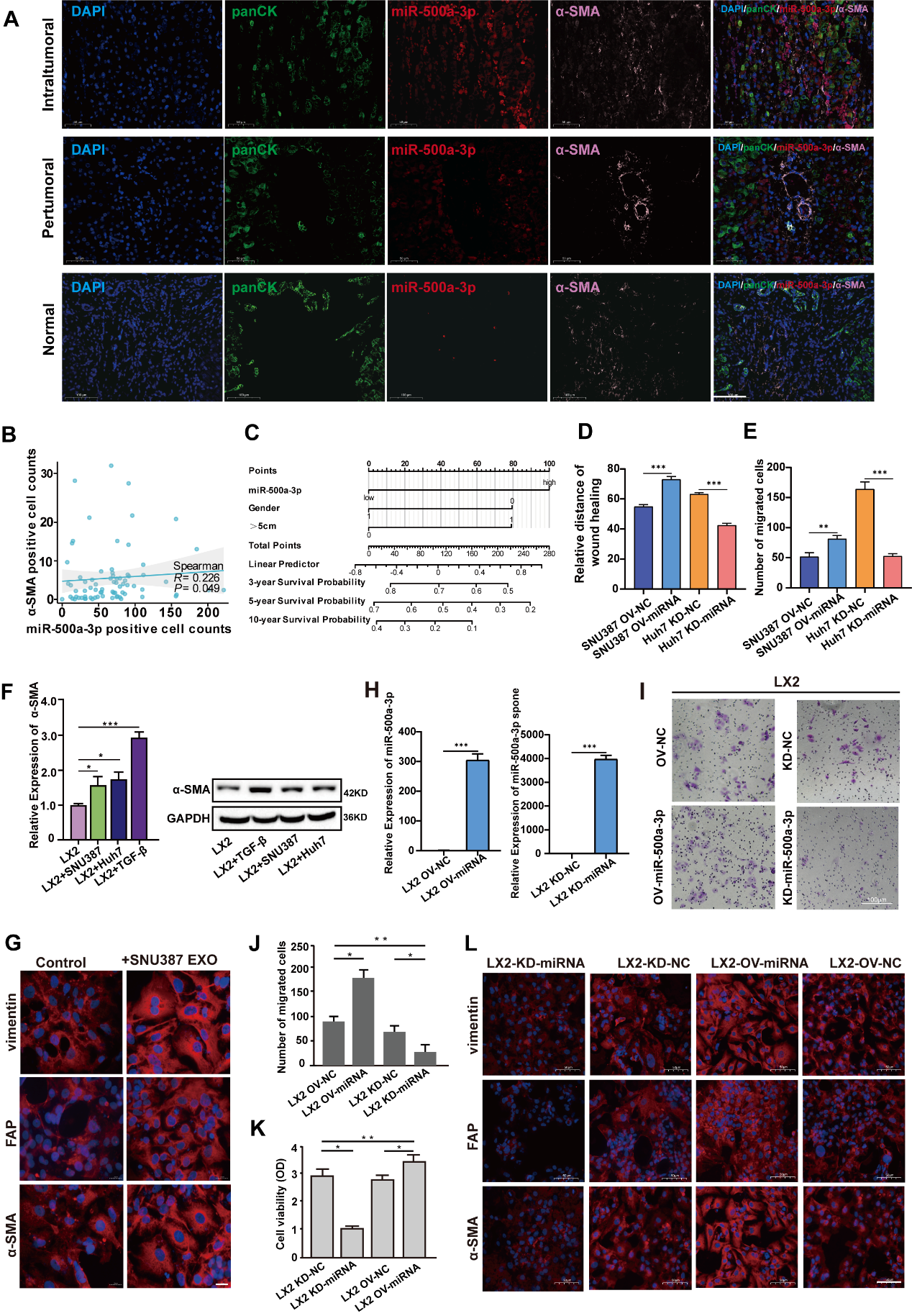
**Figure S2**. MiR-500a-3p promotes HCC progression through HSC activation and tumor cell migration. The mIHC images in the sections show intratumoral, peritumoral, and normal tissues after co-expression of α-SMA (fluorescence pink) and pan-CK (fluorescence green) with miR-500a-3p (fluorescence red and RGB blue; bar value = 100 μm). (B) The scatter diagram indicates that miR-500a-3p expression in cirrhosis positively correlates with α-SMA (n=69). (C) Prognostic nomogram used to establish the relationship between the variables in the prediction model (n=158). D-E: Wound healing assay (D) and transwell assay (E) to detect the effects of miR-500a-3p on the migration ability of SNU387 and Huh-7 cell lines. (F) RT-qPCR and Western blot verified the expression of α-SMA after co-culture of LX2 and hepatocellular carcinoma cells, and the positive control was stimulated by TGF-β1 (5 mg/mL; n=3). (G) Immunofluorescence staining to determine the effect of SUN387 exosomes on hepatic stellate cell activation (bar value = 25 μm). (H) A lentiviral vector for miR-500a-3p-OV or miR-500a-3p-KD was successfully constructed to transfect LX2 cells (n=3). (I, J) Transwell assay to detect the effect of miR-500a-3p-OV or miR-500a-3p-KD on the migration of LX2 cells (n=3). (K) CCK-8 assay to detect the effect of miR-500a-3p on the proliferation of LX2 cells (n=3). (L) Immunofluorescence staining to detect the effect of miR-500a-3p-OV or miR-500a-3p-KD on the activation of LX2 cells (bar value = 50 μm). Data were statistically analyzed using unpaired two-tailed Student’s t-tests (H), Spearman correlation (B), or one-way ANOVA (D, E, J, K). Data are presented as the mean ± SD, *p < 0.05, **p < 0.01, ***p < 0.001.

x


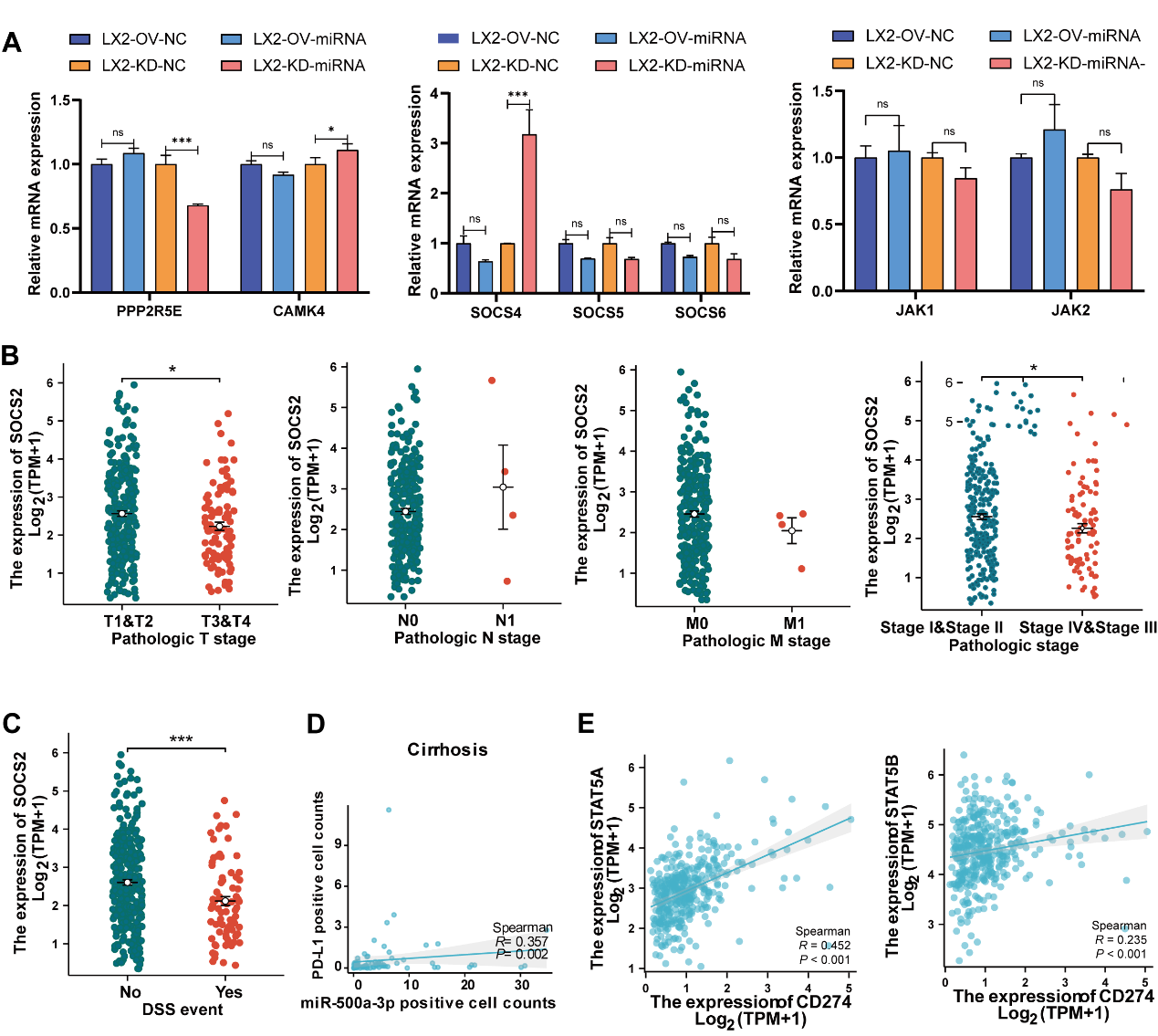


**Figure S3.** MiR-500a-3p regulates SOCS2 and JAK/STAT signaling in HCC progression. (A) qRT-PCR analyses of the relative levels of *PPP2R5E*, *CAMK4*, *SOCS4*, *SOCS5*, *SOCS6*, *JAK1*, and *JAK2* expression in LX2 cells after transfection with miR-500a-3p-KD or miR-500a-3p-OV (n=3). (B) Bioinformatic analysis of the expression of *SOCS2* using the TCGA database. (C) Relationship between *SOCS2* and Disease-free survival in patients with HCC within the TCGA cohort. (D) The scatter diagram indicates that the miR-500a-3p expression in cirrhosis samples was positively correlated with PD-L1 expression in cirrhosis samples. (E) The scatter diagram indicates that the STAT5A/STAT5B expression was positively correlated with *SOCS2* in the TCGA LIHC cohort. Data were statistically analyzed using one-way ANOVA (A) or Spearman correlation (D, E). Data are presented as mean ± SD, ns > 0.05, *p < 0.05, **p < 0.01, ***p < 0.001.


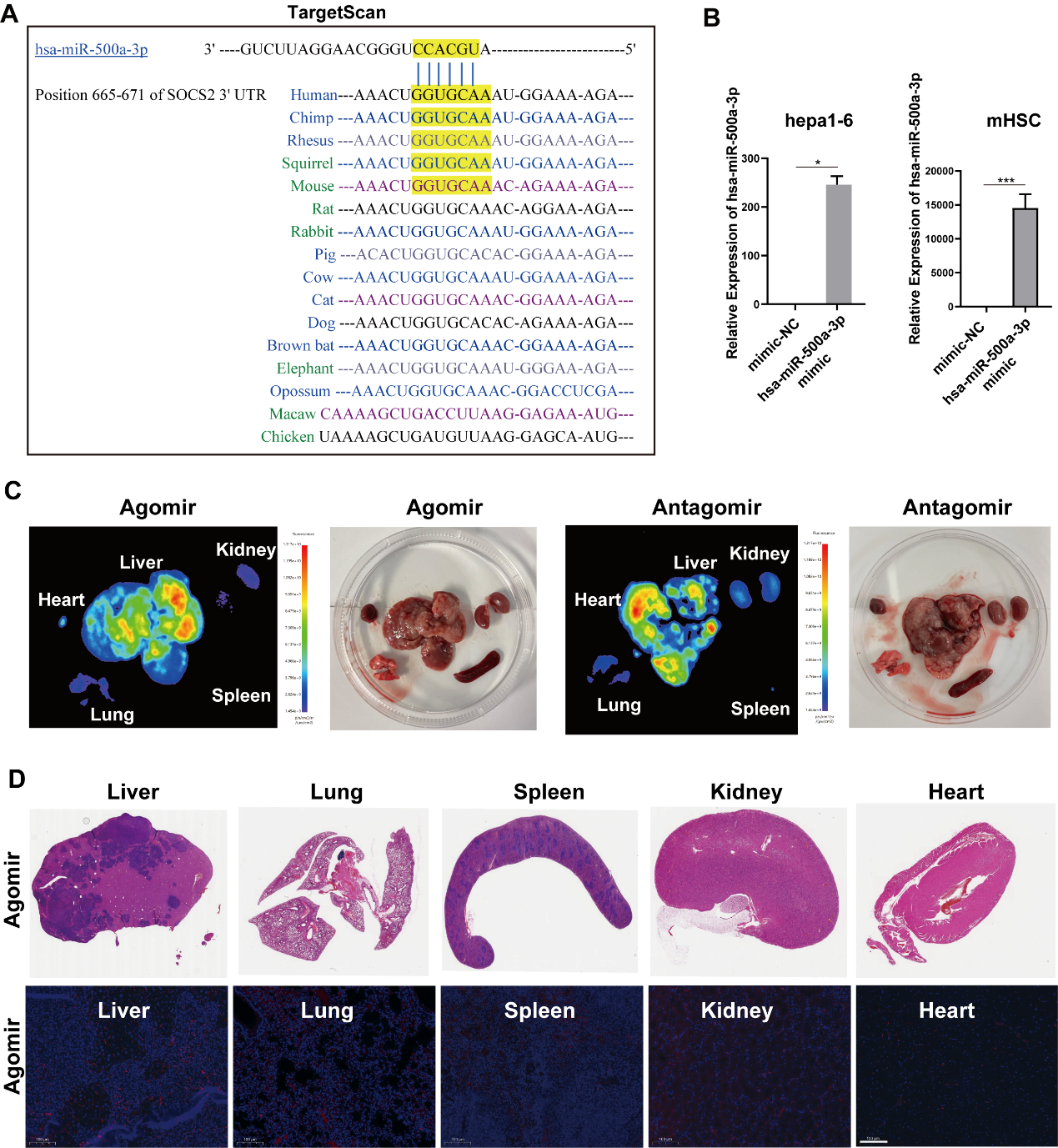


**Figure S4.** Characterization and *in-vivo* distribution of miR-500a-3p. (A) Conservation of miR-500a-3p binding sites in SOCS2 3′UTR in vertebrates. SOCS2 3′UTR from different species are aligned together with miR-500a-3p. The seed region and its matching nucleotides are indicated in yellow. Base pairing is indicated by a line. (B) To verify the effects of hsa-miR-500a-3p, we overexpressed or knocked down hsa-miR-500a-3p in the mouse hepatocellular carcinoma cell line Hepa1-6 and mouse hepatic stellate cells (mHSCs), respectively. RT-qPCR detected corresponding changes in hsa-miR-500a-3p expression levels (n=3). (C) Three hours after tail vein injection of agomir and antagomir, the liver, spleen, lung, brain, and heart of treated mice were harvested and imaged again using the *in-vivo* imaging system. (D) Hematoxylin and eosin and has-miR-500a-3p FISH probe staining were used to clarify the accumulation of agomir and antagomir in various mouse organs (bar value = 100 μm). Data were statistically analyzed using unpaired two-tailed Student’s t-tests (B). Data are presented as the mean ± SD, *p < 0.05, ***p < 0.001.


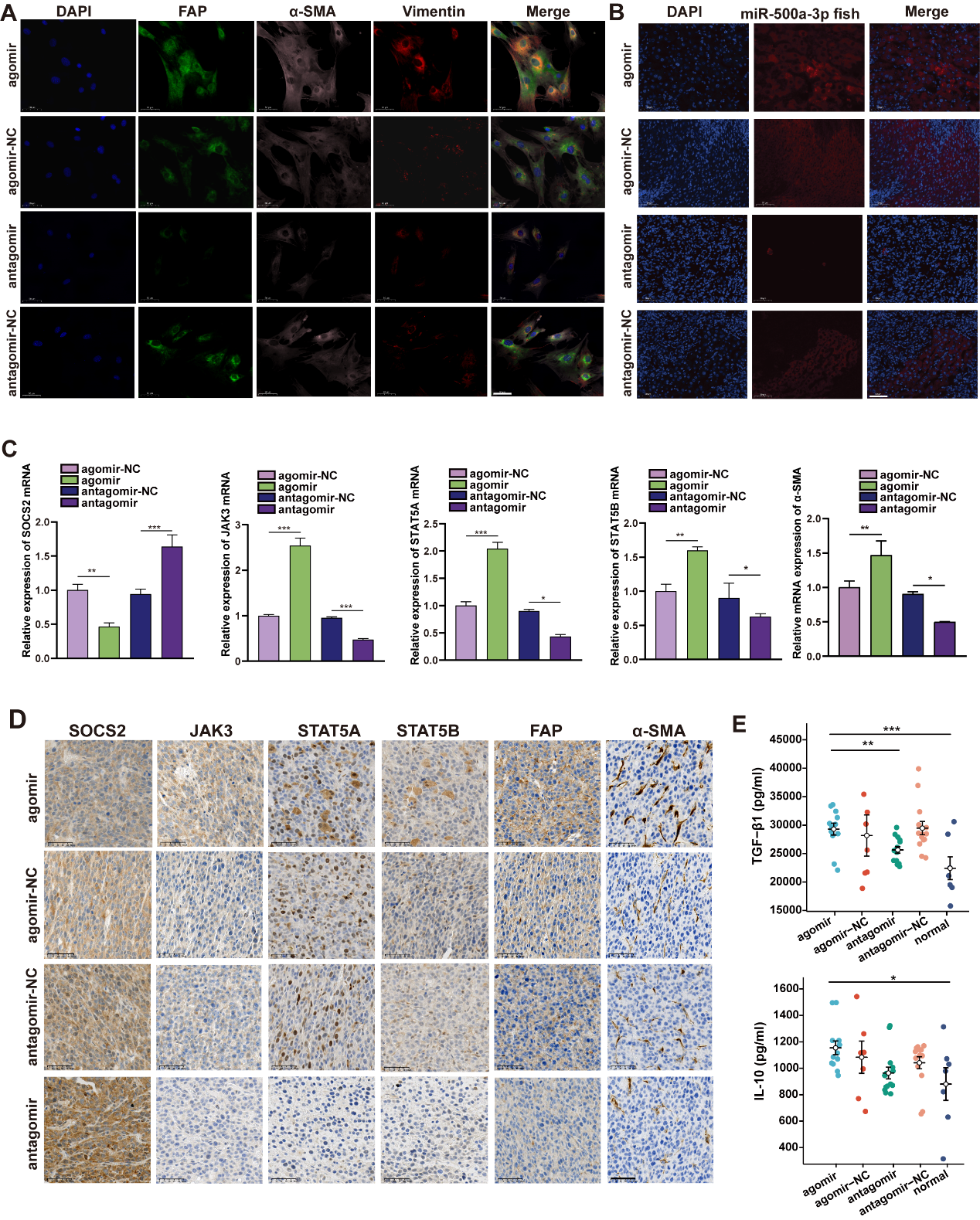


**Figure S5** MiR-500a-3p modulates HSC activation and immune suppression in *in-vivo* HCC models. (A) Multi-immunofluorescence staining of primary HSCs extracted from ICR mice to detect the effect of the agomir, antagomir, and NC groups on LX2 cell activation in the *in-situ* ICR HCC model (bar value = 50 μm). (B) Expression of miR-500a-3p fish probe staining of four groups in the *in-situ* ICR HCC model (bar value = 50 μm). (C) RT-qPCR to determine the levels of SOCS2/JAK3/STAT5A/STAT5B and α-SMA expression in the four groups (n=3). (D) Representative images of IHC staining of xenograft tumor revealed the effects of miR-500a-3p on the SOCS2/ STAT5A/STAT5B axis, and the activating α-SMA and FAP (bar value = 50 μm). (E) ELISA to detect the level of TGF-β1 and IL-10 in the miR-500a-3p of the agomir, agomir-NC, antagomir, antagomir-NC, and normal groups of *in-situ* HCC ICR mouse models (n=6). Data were statistically analyzed using one-way ANOVA. Data are presented as the mean ± SD, *p < 0.05, **p < 0.01, ***p < 0.001.

**Table S1. Clinicopathological features of HCC patients with differential expression of miR-500a-3p**

| Characteristics | hsa-miR500a-3p (low) | hsa-miR500a-3p (high) | P-value |
| --- | --- | --- | --- |
| N | 91 | 67 |  |
| Sex n (%) |  |  | 0.121 |
| 0 | 48 (30.4%) | 27 (17.1%) |  |
| 1 | 43 (27.2%) | 40 (25.3%) |  |
| Age, n (%) |  |  | 0.176 |
| 0 | 65 (41.1%) | 41 (25.9%) |  |
| 1 | 26 (16.5%) | 26 (16.5%) |  |
| T stage, n (%) |  |  | 0.321 |
| T1+T2 | 81 (51.3%) | 56 (35.4%) |  |
| T3+T4 | 10 (6.3%) | 11 (7%) |  |
| N stage, n (%) |  |  | 0.917 |
| N0 | 84 (53.2%) | 63 (39.9%) |  |
| N1 | 7 (4.4%) | 4 (2.5%) |  |
| M stage, n (%) |  |  | 0.841 |
| M0 | 88 (55.7%) | 66 (41.8%) |  |
| M1 | 3 (1.9%) | 1 (0.6%) |  |
| Tumor size > 5 cm, n (%) |  |  | 0.571 |
| ≤ 5 cm | 61 (38.6%) | 42 (26.6%) |  |
| > 5 cm | 30 (19%) | 25 (15.8%) |  |
| Number of tumors, n (%) |  |  | 0.535 |
| Single | 73 (46.2%) | 51 (32.3%) |  |
| Multiple | 18 (11.4%) | 16 (10.1%) |  |
| Vascular invasion, n (%) |  |  | 0.537 |
| No | 78 (49.4%) | 55 (34.8%) |  |
| Yes | 13 (8.2%) | 12 (7.6%) |  |
| Portal vein embolism, n (%) |  |  | 0.147 |
| No | 84 (53.2%) | 57 (36.1%) |  |
| Yes | 7 (4.4%) | 10 (6.3%) |  |
| Neuroaggression, n (%) |  |  | 0.714 |
| No | 86 (54.4%) | 65 (41.1%) |  |
| Yes | 5 (3.2%) | 2 (1.3%) |  |
| Satellite nodule, n (%) |  |  | 0.205 |
| No | 90 (57%) | 63 (39.9%) |  |
| Yes | 1 (0.6%) | 4 (2.5%) |  |
| Peripheral liver tissue cirrhosis, n (%) |  |  | 0.825 |
| No | 50 (31.6%) | 38 (24.1%) |  |
| Yes | 41 (25.9%) | 29 (18.4%) |  |

**Table S2.** Univariate and multivariate analyses of factors associated with hepatocellular carcinoma survival

| Characteristics | Total (N) | Univariate analysis | |  | Multivariate analysis | |
| --- | --- | --- | --- | --- | --- | --- |
|  |  | Hazard ratio (95% CI) | P-value |  | Hazard ratio (95% CI) | P-value |
| Has-miR-500a-3p | 158 |  |  |  |  |  |
| Low | 91 | Reference |  |  | Reference |  |
| High | 67 | 1.592 (1.102–2.301) | **0.013** |  | 1.910 (1.273–2.867) | **0.002** |
| Sex | 158 |  |  |  |  |  |
| 0 | 75 | Reference |  |  | Reference |  |
| 1 | 83 | 0.726 (0.500–1.054) | 0.092 |  | 0.611 (0.408–0.915) | **0.017** |
| Age | 158 |  |  |  |  |  |
| 0 | 106 | Reference |  |  |  |  |
| 1 | 52 | 1.081 (0.731–1.598) | 0.697 |  |  |  |
| T Stage | 158 |  |  |  |  |  |
| T1+T2 | 137 | Reference |  |  | Reference |  |
| T3+T4 | 21 | 2.112 (1.275–3.499) | **0.004** |  | 0.947 (0.365–2.458) | 0.911 |
| N Stage | 158 |  |  |  |  |  |
| N0 | 147 | Reference |  |  |  |  |
| N1 | 11 | 1.744 (0.910–3.343) | 0.094 |  |  |  |
| M Stage | 158 |  |  |  |  |  |
| M0 | 154 | Reference |  |  |  |  |
| M1 | 4 | 1.076 (0.337–3.435) | 0.902 |  |  |  |
| Tumor size > 5 cm | 158 |  |  |  |  |  |
| No | 103 | Reference |  |  | Reference |  |
| Yes | 55 | 1.713 (1.174–2.501) | **0.005** |  | 1.578 (1.064–2.339) | **0.023** |
| Number of tumors | 158 |  |  |  |  |  |
| No | 124 | Reference |  |  |  |  |
| Yes | 34 | 1.218 (0.790–1.876) | 0.372 |  |  |  |
| Vascular invasion | 158 |  |  |  |  |  |
| No | 133 | Reference |  |  |  |  |
| Yes | 25 | 1.531 (0.896–2.619) | 0.119 |  |  |  |
| Portal vein embolism | 158 |  |  |  |  |  |
| No | 141 | Reference |  |  | Reference |  |
| Yes | 17 | 2.292 (1.333–3.940) | **0.003** |  | 1.898 (0.698–5.162) | 0.209 |
| Neuroaggression | 158 |  |  |  |  |  |
| No | 151 | Reference |  |  |  |  |
| Yes | 7 | 0.570 (0.231–1.411) | 0.225 |  |  |  |
| Satellite nodule | 158 |  |  |  |  |  |
| No | 153 | Reference |  |  |  |  |
| Yes | 5 | 1.208 (0.445–3.284) | 0.710 |  |  |  |
| Peripheral liver tissue cirrhosis | 158 |  |  |  |  |  |
| No | 88 | Reference |  |  | Reference |  |
| Yes | 70 | 0.727 (0.499–1.059) | 0.097 |  | 0.747 (0.502–1.110) | 0.149 |

**Table S3. Primer sequences used for qRT-PCR**

| Name | Sense primer | Anti-sense primer |
| --- | --- | --- |
| α-SMA | GGGGTGATGGTGGGAATG | GCAGGGTGGGATGCTCTT |
| FAP- | TATTCCATACCCAAAGGC | GACAGGACCGAAACATTC |
| Vimentin | TTGAACGCAAAGTGGAATC | AGGTCAGGCTTGGAAACA |
| FSP1 | GCCCTGGATGTGATGGTG | CGTTGTCCCTGTTGCTGT |
| JAK1 | GTGGAGGTAACCACATAGC | CCGAGAACCCAAATAGTC |
| JAK2 | GCCTTCTTTCAGAGCCATCA | TTACAGCGACCACCTCCC |
| JAK3 | AGCCCTTCTGCGACTTTCC | ATCTGGTTGTCTGTCCTGGTAA |
| STAT5A | GAGAACACCCGCAACGAG | CCGTGGACGATGACAACC |
| STAT5B | TTCAGTGTTGGTGGAAATG | TCTGGCTTGTTAATGAGTAGG |
| SOCS2 | GCTCGCATTCAGACTACCT | CCTGTCCGCTTATCCTTG |
| SOCS4 | AGGTCCTATGACTGGCTCTG | TTCGGCTGCGTATTTATC |
| SOCS5 | GCTCCTGGAATGACTGAA | TAACATGGGTATGGCTGT |
| SOCS6 | GATGATGTCCCTCCACTC | GGTAACGGTCGTCAGAAC |

Supporting Data 1: This image represents the original data used to generate the results presented in Figure 2L and 3I.


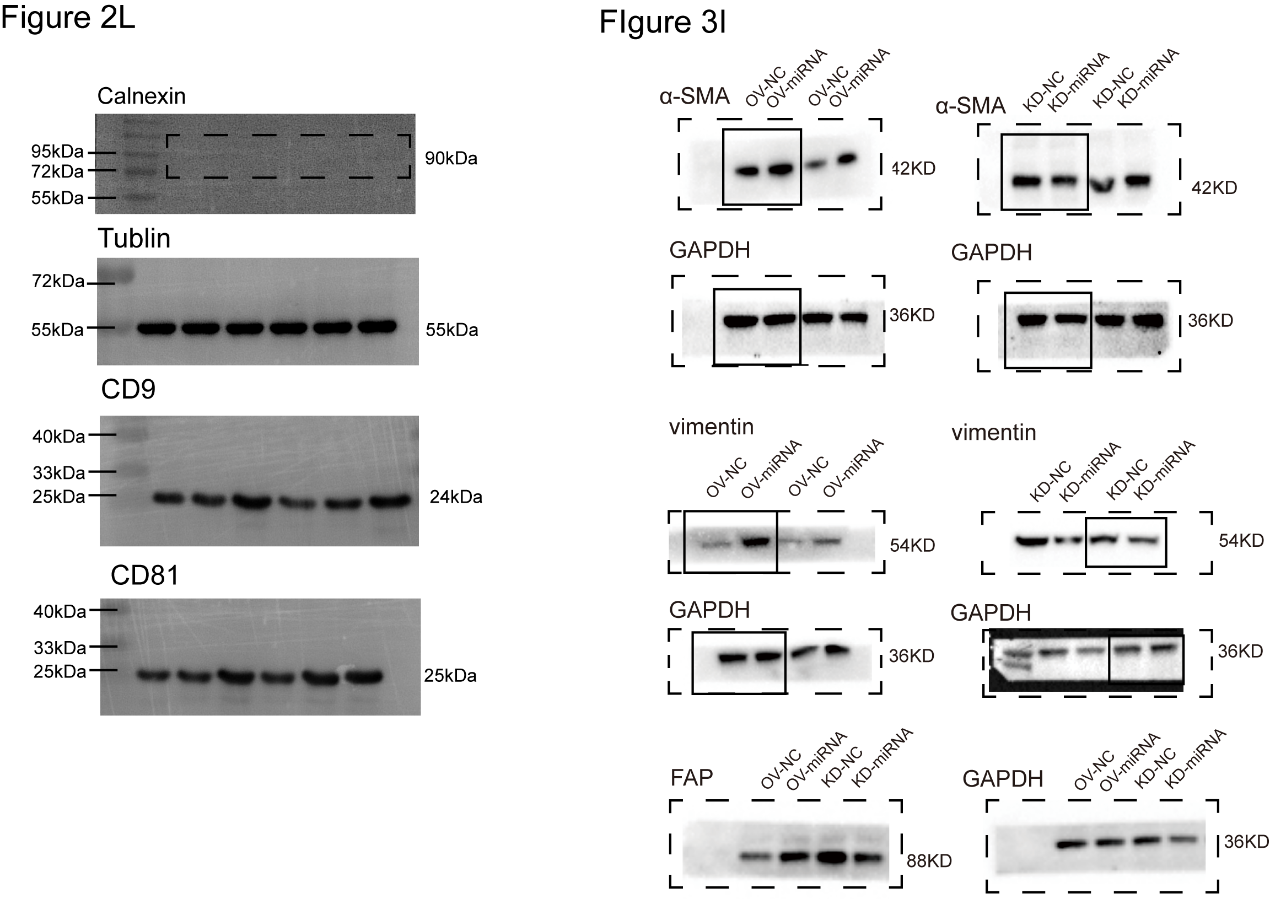


Supporting Data 2: This image represents the original data used to generate the results presented in Figure 4I and 4J .


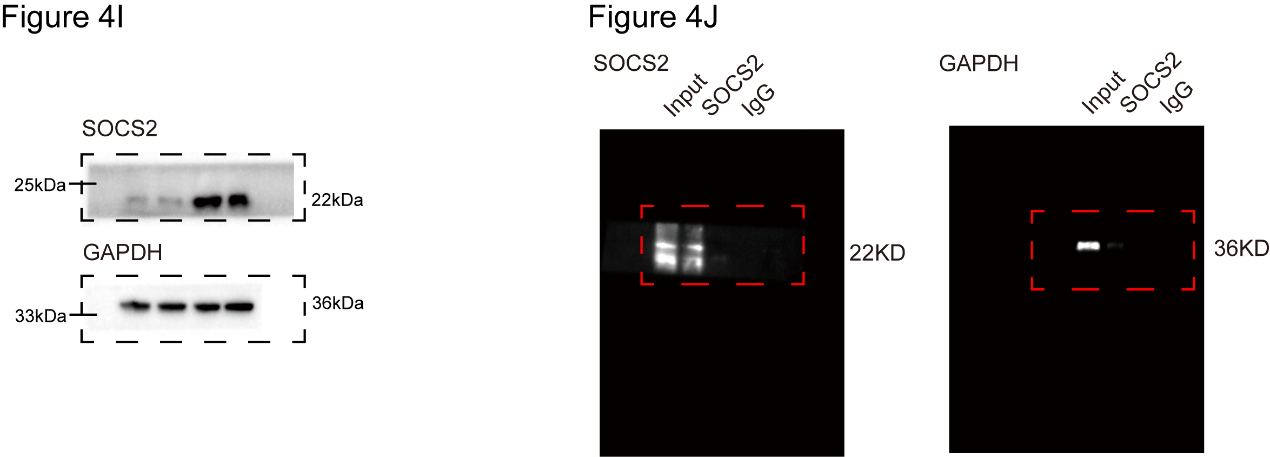


Supporting Data 3: This image represents the original data used to generate the results presented in Figure 4O, 5C and 6K.


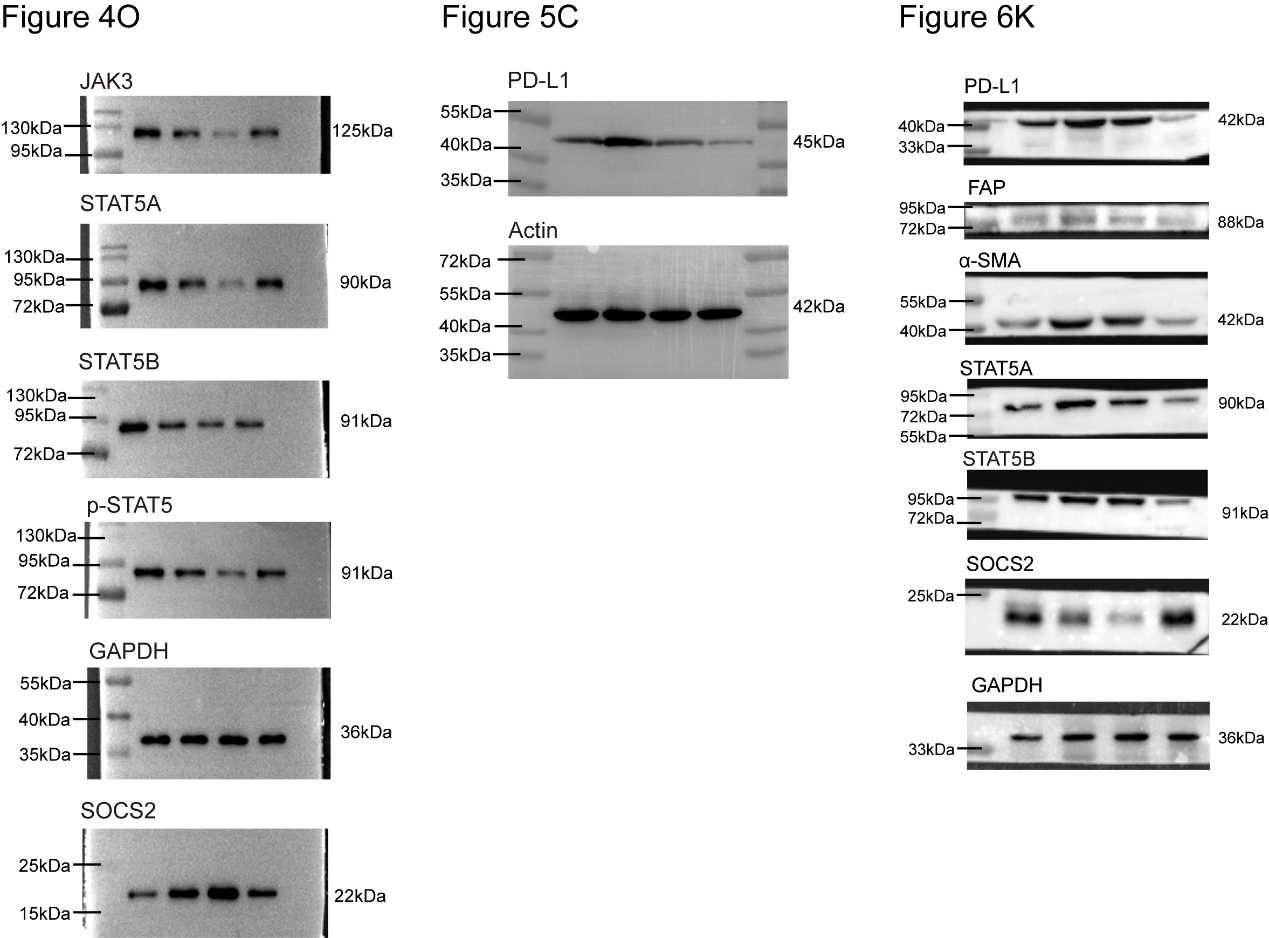


Supporting Data 4: This image represents the original data used to generate the results presented in Figure 6C.


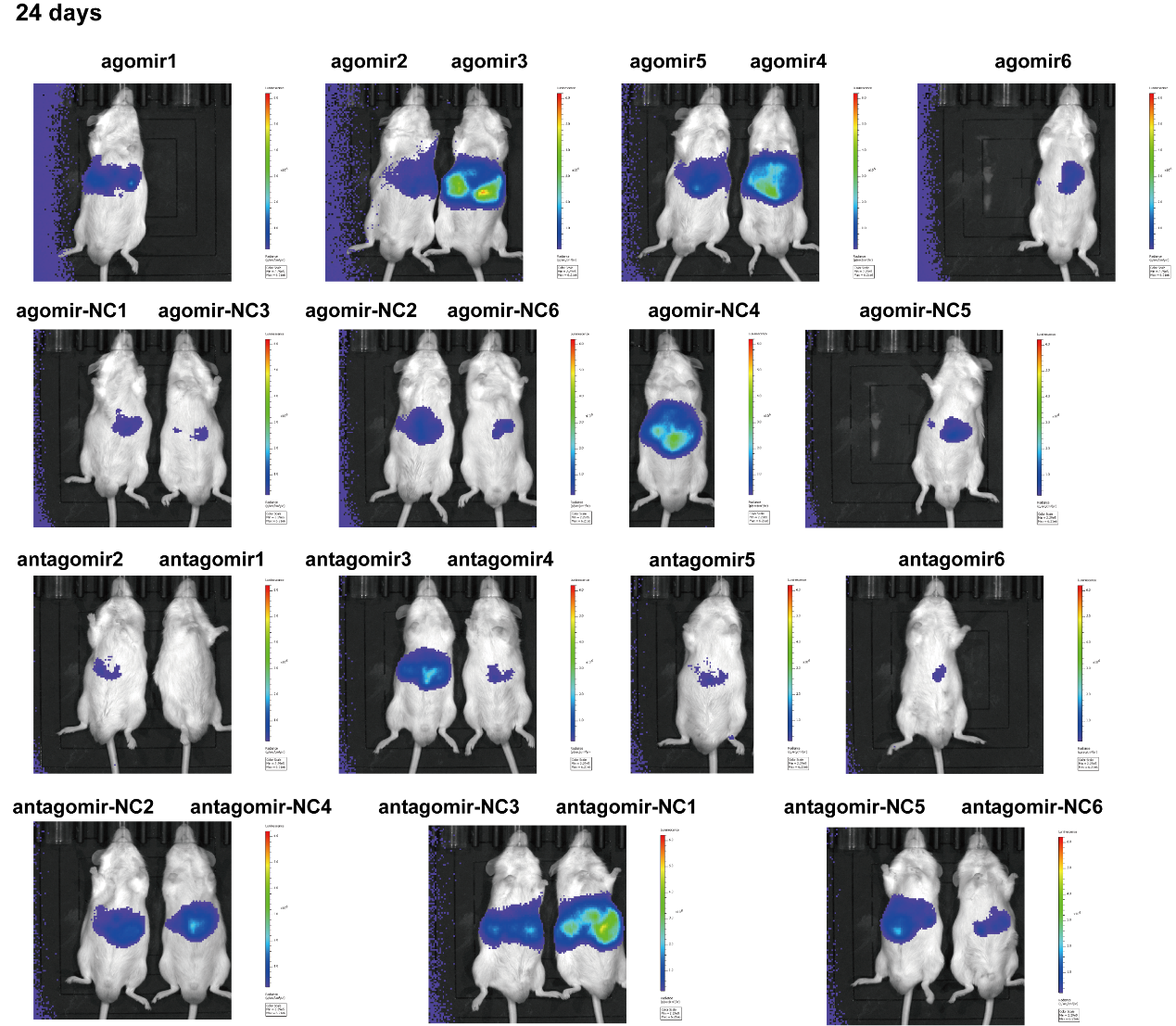

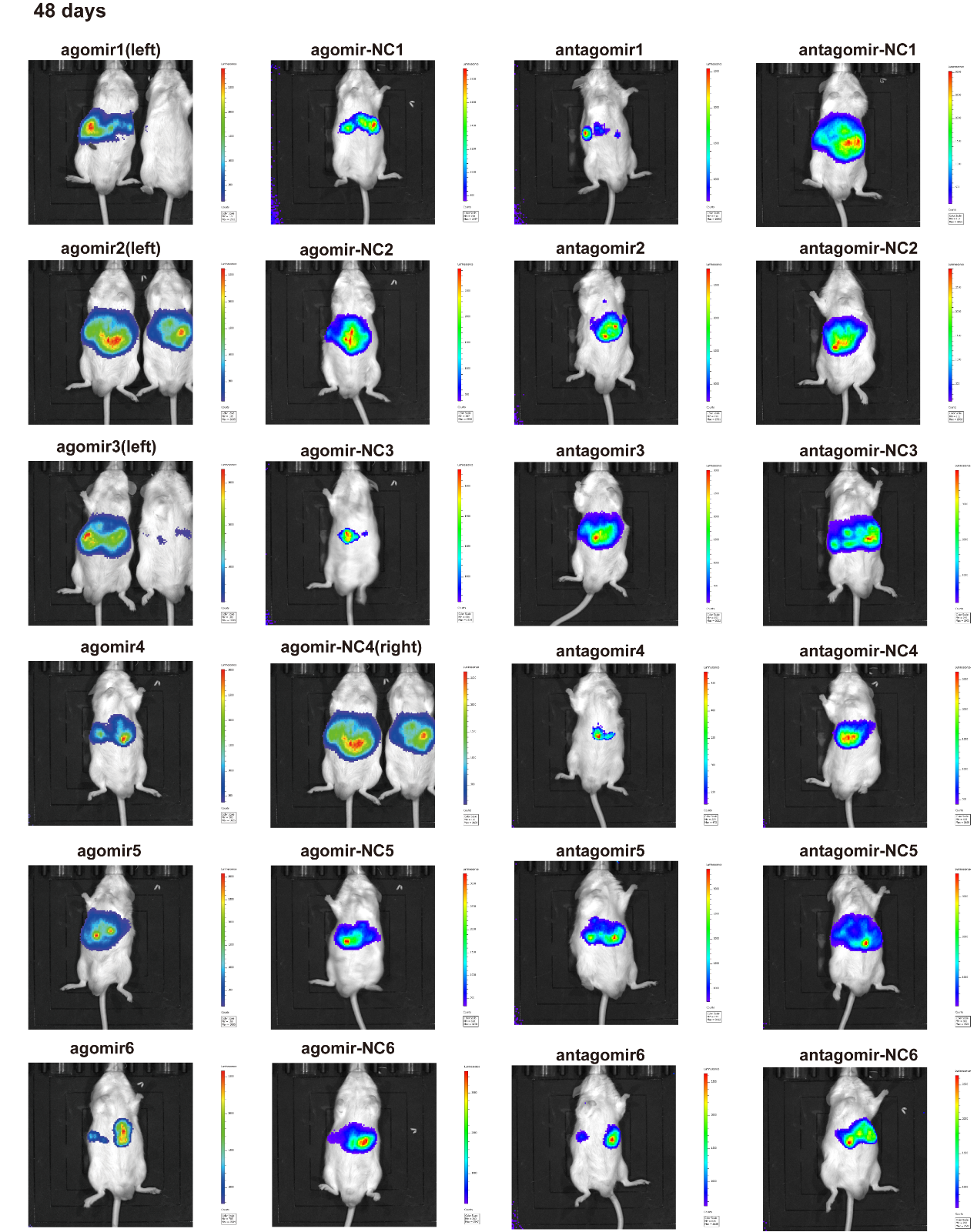

Supplement: Supplementary file 1 — Supporting Information [file ADVS-12-2404089-s001.docx]
